# The efficacy and safety of first-line monotherapies in primary therapy of invasive aspergillosis: a systematic review

**DOI:** 10.3389/fphar.2024.1530999

**Published:** 2025-01-15

**Authors:** Yan Chen, Jiaojiao Zhao, Yifei Wang, Long Ge, Joey Sum-wing Kwong, Junjie Lan, Rui Zhang, Huaye Zhao, Linfang Hu, Jiaxue Wang, Shuimei Sun, Songsong Tan, Xiaoqing Lin, Rui He, Wenyi Zheng, Xiaosi Li, Jiaxing Zhang

**Affiliations:** ^1^ School of Pharmaceutical Sciences, Guizhou University, Guiyang, China; ^2^ Department of Pharmacy, Guizhou Provincial People’s Hospital, Guiyang, China; ^3^ School of Traditional Chinese Medicine, Tianjin University of Traditional Chinese Medicine, Tianjin, China; ^4^ Evidence-Based Social Science Research Center, School of Public Health, Lanzhou University, Lanzhou, China; ^5^ Department of Social Medicine and Health Management, School of Public Health, Lanzhou University, Lanzhou, China; ^6^ Global Health Nursing, Graduate School of Nursing Science, St. Luke’s International University, Tokyo, Japan; ^7^ Office of Health Insurance Administration, Guizhou Provincial People’s Hospital, Guiyang, China; ^8^ School of Public Health, The key Laboratory of Environmental Pollution Monitoringand Disease Control, Ministry of Education, Guizhou Medical University, Guiyang, China; ^9^ Experimental Cancer Medicine, Department of Laboratory Medicine, Karolinska Institute, Stockholm, Sweden; ^10^ Department of Pharmacy, Hospital of Chengdu Office of People's Government of Tibetan Autonomous Region, Chengdu, China

**Keywords:** invasive aspergillosis, antifungals, network meta-analysis, aspergillus, posaconazole, amphotericin B deoxycholate, isavuconazole, voriconazole

## Abstract

**Objective:**

Several antifungals are available for the treatment of patients with invasive aspergillosis (IA). This study aims to evaluate the relative efficacy and safety of the first-line monotherapies in primary therapy of IA through network meta-analysis (NMA).

**Methods:**

We systematically searched PubMed, Embase, Web of Science, Cochrane Library, China National Knowledge Infrastructure, VIP database, Wanfang database, and China Biology Medicine for randomized controlled trials (RCTs) up to July 2023 that evaluated the efficacy and safety of monotherapies. We performed NMA with a frequentist random effects model and assessed the certainty of evidence using the GRADE (Grading of Recommendations Assessment, Development and Evaluation) approach. Primary outcomes were the all-cause mortality at week 12, and secondary outcomes included overall response rate, and incidence of adverse events (AEs) and severe adverse events (SAEs).

**Results:**

A total of three RCTs involving 1,368 participants (four antifungals) were included. The NMA showed that compared to amphotericin B deoxycholate (D-AmB), the triazoles (posaconazole (POS), isavuconazole (ISA) and voriconazole (VCZ)) can improve the overall response rate in primary therapy of IA, but only VCZ and ISA can reduce the all-cause mortality at week 12 for patients with proven and probable IA (VCZ vs D-AmB: RR = 0.66, 95%CI = 0.47–0.93, moderate certainty; ISA vs D-AmB: RR = 0.52, 95%CI = 0 .31–0.86, low certainty). ISA (SUCRA = 93.50%; mean rank, 1.20) seemed to be the most effective therapy in the above population. As to proven, probable, and possible IA patients, the triazoles were superior to D-AmB in terms of reducing all-cause mortality. Furthermore, the risk of AEs and SAEs was comparable for the three triazoles, but the risk of SAEs was significantly higher for D-AmB than others.

**Conclusion:**

The efficacy and safety of triazoles are more favorable than D-AmB in the primary therapy of IA, with ISA being the optimal choice.

**Systematic Review Registration:**

PROSPERO CRD42023407632.

## 1 Introduction

Invasive aspergillosis (IA) is a life-threatening invasive fungal infection caused by Aspergillus fungi, with over 300,000 cases worldwide annually (Bongomin et al., 2017) and a mortality of 80%–90% among high-risk patients (Del Bono et al., 2008). IA includes invasive pulmonary aspergillosis (IPA), Aspergillus sinusitis, disseminated aspergillosis, and several types of single-organ IA, and is usually seen in immunocompromised populations such as those with prolonged neutropenia, allogeneic hematopoietic stem cell transplantation (HSCT) (Patterson et al., 2000), solid organ transplant (SOT) (Husain and Camargo, 2019), inherited or acquired immunodeficiencies (Baddley, 2011), corticosteroid use (Shi et al., 2022), and others.

According to the clinical contexts, IA can be divided into primary IA where the patient is not exposed to a mould-active antifungal at presentation or within the last 7 days), breakthrough IA, and refractory IA (Cornely et al., 2019). In 2008, the European Organization for Research and Treatment of Cancer Mycoses Study Group (EORTC/MSG) categorized the likelihood of diagnosing IA into proven, probable, possible, and uncertain or not aspergillosis (De Pauw et al., 2008). Therefore, the primary therapy of IA was defined as the first therapy used upon diagnosis or suspicion of an IA. The 2016 Infectious Diseases Society of America (IDSA) guideline (Patterson et al., 2016) recommended voriconazole (VCZ) as primary therapy for both IPA and extrapulmonary aspergillosis. Then, the European Conference on Infection in Leukemia (ECIL)-6 guideline (Frederic et al., 2017), the 2017 European Society for Clinical Microbiology and Infectious Diseases (ESCMID)-European Confederation of Medical Mycology (ECMM)-European Respiratory Society (ERS) guideline (Ullmann et al., 2018), and the 2018 Study Group of Fungal Infections from the Spanish Society of Infectious Diseases and Clinical Microbiology (GEMICOMED-SEIMC/REIPI) guideline (Garcia-Vidal et al., 2019) endorsed both VCZ and isavuconazole (ISA) as equally effective choices for treating IA in hematological patients and recommended them as the first-line therapies. Posaconazole (POS), a broad-spectrum triazole (Patterson et al., 2016; Frederic et al., 2017), was approved by the Food and Drug Administration (FDA) for prophylaxis or salvage therapy of IA in adults and pediatric patients 13 years of age and older in 2006 (Assasi and Grobelna, 2017). Furthermore, 2021 consensus guidelines for the diagnosis and management of IA (Douglas et al., 2021) also recommended POS as an alternative to VCZ for treating IPA in hematology/oncology patients. With VCZ, ISA, and POS being highly anticipated as monotherapies, clinicians are increasingly focused on comparing their relative efficacy and safety in the primary therapy of IA.

At present, most network meta-analysis (NMA) (Bow et al., 2015; Lee et al., 2018; Leonart et al., 2017; Marinelli et al., 2022; Su et al., 2019; Wang et al., 2020) focus on the prophylaxis of IA, with a few studies addressing primary treatment. For instance, Herbrecht et al. (2018) conducted a systematic review and NMA to estimate the relative efficacy of ISA compared with amphotericin B (AmB) deoxycholate, liposomal AmB, and VCZ for the treatment of patients with proven and probable IA, but this study did not include POS in the analyses. Similarly, Liu et al. (2024) performed a NMA to compare the efficacy of different antifungals, including both monotherapy and combination therapy, in IA. Nonetheless, current guidelines (Patterson et al., 2016; Frederic et al., 2017; Garcia-Vidal et al., 2019; Douglas et al., 2021) did not routinely recommend combination therapy for primary therapy of IA. Additionally, this study included randomized controlled trials (RCTs) and observational studies in the same NMA, introducing methodological heterogeneity that may affect the robustness of the conclusions. In the absence of a study comparing the efficacy and safety of these first-line monotherapies in primary therapy of IA, we conducted this systematic review and NMA to identify the optimal treatment.

## 2 Materials and methods

### 2.1 Protocol and registration

This study is reported following the Preferred Reporting Items for Systematic Reviews and Meta-Analyses (PRISMA) NMA Checklist (eTable 1 in the Supplementary Material) (Liberati et al., 2009; Hutton et al., 2015), and the protocol has been registered in PROSPERO (CRD42023407632).

### 2.2 Search strategy

PubMed, Embase, Web of Science, Cochrane Library, The China National Knowledge Infrastructure (CNKI), VIP database, Wangfang database, and China Biology Medicine (CBM) were searched using the search strategies detailed in eTable 2 in the Supplementary Material. To collect all published RCTs regarding antifungals in the primary therapy of IA from their inception to 21 July 2023. We also manually searched for references of included studies and reviews to prevent overlooking any pertinent evidence.

### 2.3 Inclusion and exclusion criteria

We included studies meeting the following criteria: (1) RCTs; (2) Patients diagnosed with proven, probable, or possible IA whose definitions are mainly based on the revised EORTC/MSG consensus (eTable 3 in the Supplementary Material) (De Pauw et al., 2008), regardless of age or gender; (3) receiving one of the following monotherapy in the primary therapy of IA: amphotericin B (AmB), azoles (fluconazole, VCZ, itraconazole, POS, ISA), and echinocandins (micafungin, caspofungin, anidulafungin); (4) reporting on any of the following outcomes: all-cause mortality at week 12 (primary outcome); overall response rate, defined as a complete or partial response at week 12 or end of treatment (as assessed by the data review committee and the definition is the last day of study drug administration); incidence of overall adverse events (AEs); and incidence of severe adverse events (SAEs); (5) published in English or Chinese.

We excluded studies investigating various dosages or formulations of the same antimicrobial drug and studies reported as in-conference abstracts which made it impossible to assess the risk of bias.

### 2.4 Study selection and data extraction

Titles and abstracts were screened independently by two reviewers for full-text review. The full-texts of all potentially relevant articles were downloaded for further review. Discrepancies were resolved by a third reviewer.

We used a pre-designed data collection form to extract data from each eligible study, including (1) authors, year of publication, country or region where the study conducted; (2) study design; (3) antifungal used in treatment or control group, dose, administration route, and duration of treatment; (4) number of participants randomized into each group; (5) diagnosis, inclusion and exclusion criteria, gender and age of participants; (6) length of follow up; (7) outcome data (all-cause mortality at week 12, overall response rate, the number of AEs or SAEs); and (8) sources of funding. If the data in full-text and protocol on clinicalTrials.gov were inconsistent, we extracted the data from the protocol.

### 2.5 Quality assessment

Two evaluators employed the Cochrane Risk of Bias Assessment Tool (Cochrane Handbook (Higgins and Green, 2008)) to assess the methodological quality of the studies. This tool categorizes bias ratings as low, high, or unclear within six different domains, including randomization, allocation concealment, blinding of participants and outcome assessors, incomplete outcome data, and selective reporting. Although some of the studies had a high risk of bias, none was excluded from further analysis due to the small number of eligible studies (only three).

### 2.6 Certainty of evidence assessment

According to GRADE guidance, the certainty of direct evidence in randomized controlled trials is initially high and can be downgraded according to the risk of bias, indirectness, imprecision, and publication bias (Guyatt et al., 2008; Izcovich et al., 2023). Evidence from indirect comparisons could be further rated down for intransitivity. A contribution matrix quantified the proportional contribution of each direct comparison with each indirect and network comparison using the shortest path approach to determine the initial certainty of indirect evidence (Papakonstantinou et al., 2018). The final certainty for network evidence was rated down for incoherence, imprecision, or inconsistency (heterogeneity). Each domain was rated as no concern (not downgraded), some concerns (downgraded one level), and major concerns (downgraded two levels), and the certainty of the evidence of each comparison was rated as high, moderate, low or very low. Details are shown in eTable 4 in the Supplementary Material.

### 2.7 Statistical analysis

For the all-cause mortality at week 12, we respectively analyzed proven/probable/possible and proven/probable populations. The overall response rate was analyzed based on the proven/probable populations. SAEs and AEs were analyzed in participants who received at least one dose of the study drug. RevMan 5.4 software was utilized to construct risk of bias graphs for assessing the methodological quality of the included studies. We compared different antifungals through NMA performed under a frequentist framework using a random-effects model. The analysis was performed using the network and mvmeta packages in Stata statistical software version 14.0 (StataCorp). The estimated values of the results were presented as relative risk (*RR*) with their corresponding 95% confidence intervals (*CI*). *P* ≤ 0.05 was deemed to be statistically significant. The reliability and validity of the networks were estimated by addressing the heterogeneity and inconsistency in the evidence from comparative studies of different treatments. We used chi-square test with a 10% level of statistical significance to check statistical heterogeneity. A value for *I* ^*2*^ of 50% or greater was used to denote significant heterogeneity. The node-splitting method was used to conduct the local inconsistency test for direct and indirect comparisons, with *P*< 0.05 indicating the presence of local inconsistency (reported when able to perform). The efficacy ranking of each intervention was illustrated using the SUCRA (Surface Under the Cumulative Ranking Area), where a larger area under the curve signified a more beneficial effect of that intervention. The data analysis period was from December 2023 to January 2024.

## 3 Results

### 3.1 Search results and characteristics of included studies

**FIGURE 1:**
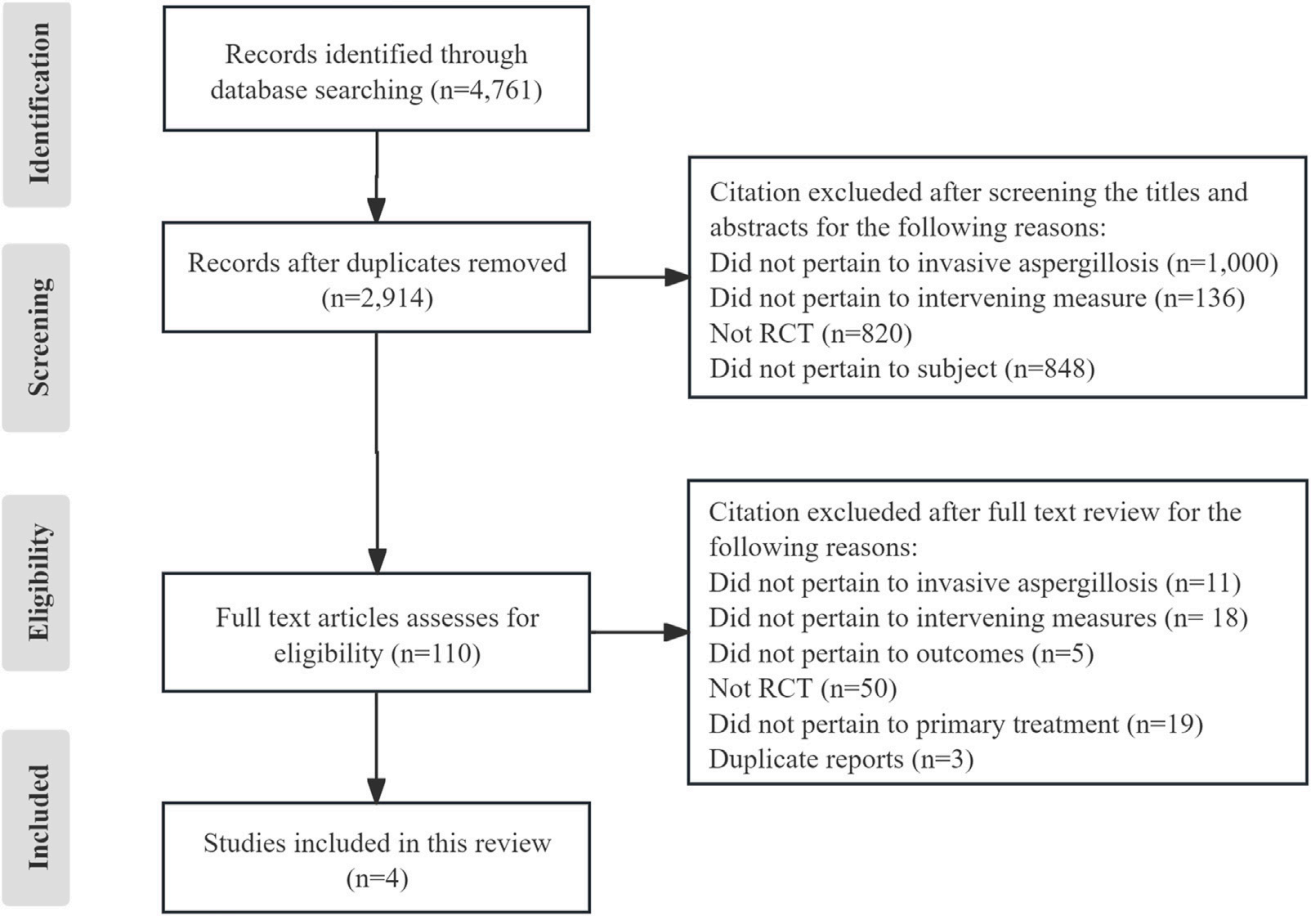
Flow diagram for studies selection. RCT: randomized controlled trials.

**FIGURE 2:**
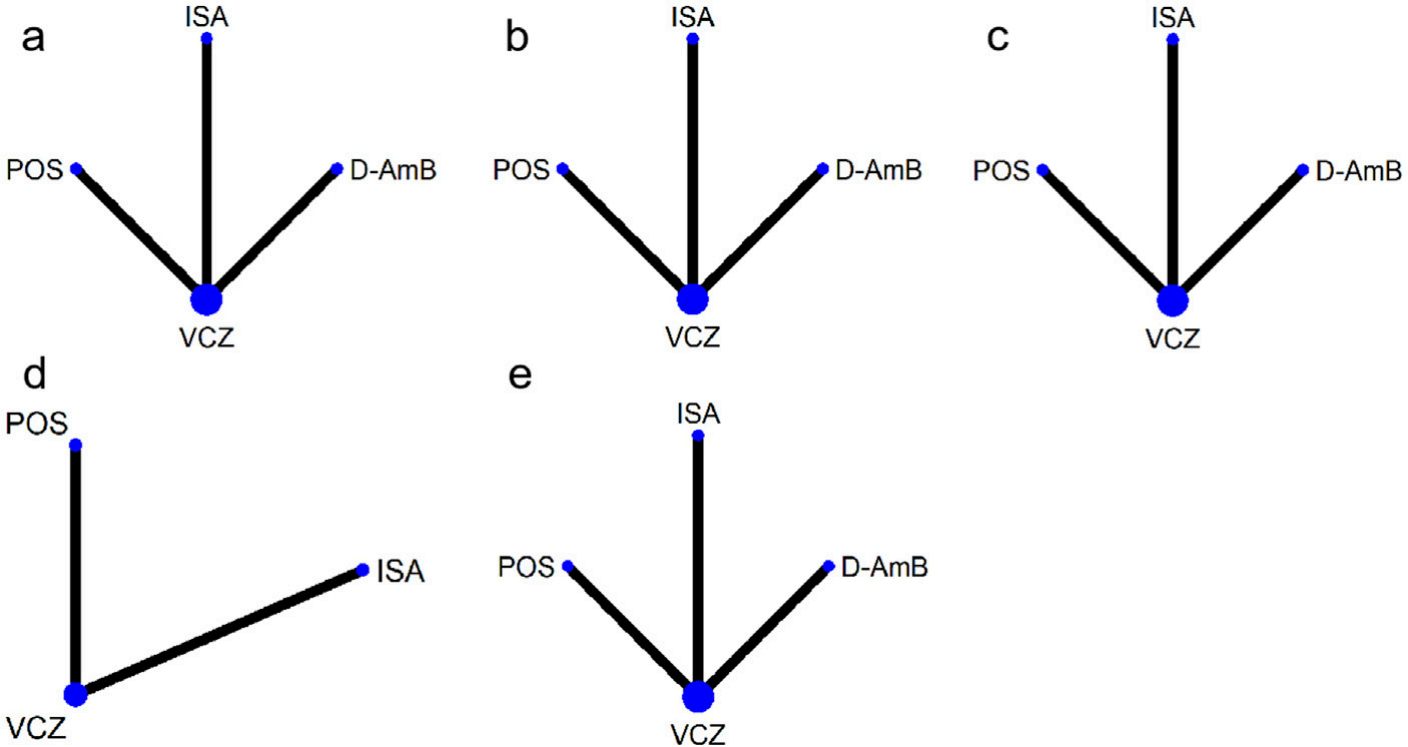
Networks of treatment comparisons for outcomes of various antifungals. The sizes of the nodes indicate the numbers of participants, and the widths of the lines indicate the numbers of included trials. **(A)** all-cause mortality at week 12 for patients with proven and probable invasive aspergillosis; **(B)** all-cause mortality at week 12 for patients with proven, probable and possible invasive aspergillosis; **(C)** overall response rate; **(D)** adverse events; **(E)** severe adverse events. VCZ: voriconazole; D-AmB: amphotericin B deoxycholate; POS: posaconazole; ISA: isavuconazole.

A total of 4,761 publications were identified from the literature search, and four (Herbrecht et al., 2002; Herbrecht et al., 2015; Maertens et al., 2021; Maertens et al., 2016) eligible publications were included in this systematic review (Figure 1). The Herbrecht 2015 study (Herbrecht et al., 2015) derived from a re-analysis of an earlier report (Herbrecht et al., 2002) due to changes in the EORTC/MSG definitions for probable and possible IA in 2008, so the two publications were considered as one study. The three studies included four interventions (Figure 2): VCZ, ISA, POS, and amphotericin B deoxycholate (D-AmB).

The baseline characteristics of the participants were shown in Table 1, which involved 1,368 participants. All studies included the IA population according to the criteria defined by the EORTC/MSG. The proportion of proven IA patients was 24.89% (59/237), 12.28% (41/334), 12.60% (65/516) in the Herbrecht 2015 study (Herbrecht et al., 2015), Maertens 2021 study (Maertens et al., 2021), Maertens 2016 study (Maertens et al., 2016), respectively. Because baseline data of proven, probable, and possible IA populations cannot be extracted from Maertens 2021 study and Maertens 2016 study, we presented the baseline data of proven, probable, possible IA, and ‘cannot be determined’ population (14.09%, 81/575) for Maertens 2021 study and of proven, probable, possible and no invasive mould disease (9.30%, 48/516) for Maertens 2016 study. Sites of IA included lung only, sinus, cerebral (category includes those with other organ involvement), disseminated (category excludes those with cerebral involvement), and others. However, the specific numbers or proportions for the infected sites were not present in two studies. The Herbrecht 2015 study did not provide information on the infected sites for the re-classified population, while the Maertens 2016 study did not provide related information for the IA patients (the mycological intention-to-treat (myITT) population). The duration of treatment was 12 weeks.

**TABLE 1:** Main characteristics of included studies.

Study Year	Registration number	Trial design	Country/Region	Proven/Probable/Possible IA	Treatment (n)	Baseline					Outcomes	Duration of treatment (weeks)
						Mean age (years, median (range))/mean ± standard deviation	Male (n (%))	Underlying disease (n (%))	Diagnostic criteria	Sites of IA		
Herbrecht et al. (2015)	*NA*	RCT	Europe, Israel, Australia, United States, Canada, Mexico, Brazil, and India	59/178/106	D-AmB (164) vs VCZ (179)	52.50 (12.00–75.00) vs 42.00 (13.00–79.00)	101 (61.58) vs 117 (65.36)	D-AmB vs VCZ Neutrophils<500/µL: 81 (49.39) vs 90 (50.28) allo-HSTC: 34 (20.73) vs 41 (22.90) auto-HSTC: 8 (4.88) vs 11 (6.15) AML: 63 (38.41) vs 64 (35.75) ALL: 12 (7.32) vs 15 (8.38) HM: 25 (15.24) vs 21 (11.73) SOC: 0 (0.00) vs 2 (1.12) SOT: 6 (3.66) vs 11 (6.15) ONMD: 16 (9.76) vs 14 (7.82)	EORTC/MSG	Lung only, Sinus Cerebral plus other organ Disseminated excluded cerebral involvement, Other	①②④	12
Maertens et al. (2021)	NCT01782131	RCT	Asia, Pacific region, Europe, North and South America	41/293/160	POS (288) vs VCZ (287)	53.50 ± 16.70 vs 53.00 ± 15.90	172 (59.72) vs 172 (59.93)	POS vs VCZ Neutropenia: 179 (62.15) vs 189 (65.85) allo-HSTC: 65 (22.60) vs 59 (20.56) TIS: 126 (43.75) vs 109 (37.98) CT: 93 (32.29) vs 89 (31.01) ISID: 2 (0.69) vs 1 (0.35) None of the above: 17 (5.90) vs 18 (6.27)	EORTC/MSG	Lung Multiple sites Sinus Other	①②③④	12
Maertens et al. (2016)	NCT00412893	RCT	North and South America, Europe, the Middle East, southeast Asia, east Asia, Pacific regions	65/207/196	ISA (258) vs VCZ (258)	51.10 ± 16.20 vs 51.20 ± 15.90	145 (56.20) vs 163 (63.18)	ISA vs VCZ AML: 99 (38.37) vs 126 (48.84) ALL: 30 (11.63) vs 24 (9.30) Lymphoma: 33 (12.79) vs 24 (9.30) MDS: 23 (8.91) vs 14 (5.43) CLL: 10 (3.88) vs 13 (5.04) AA: 9 (3.49) vs 7 (2.71) CML: 5 (1.94) vs 8 (3.10) MM: 5 (1.94) vs 7 (2.71) COPD: 5 (1.94) vs 3 (1.16) HD: 2 (0.78) vs 3 (1.16) DM: 4 (1.55) vs 0 (0.00)	EORTC/MSG	LRTD only LRTD plus other organ Non-LRTD only	①②③④	12

IA: invasive aspergillosis; *NA*: not applicable; RCT: randomized controlled trials; D-AmB: amphotericin B deoxycholate, 1.0–1.5 mg/kg/day; VCZ: voriconazole, 8 mg/kg/day; 400 mg/day; allo-HSTC: Allogeneic hematopoietic-cell transplantation; auto-HSTC: Autologous hematopoietic-cell transplantation; AML: acute myeloid leukaemia; ALL: acute lymphoblastic leukaemia; HM: hematologic malignancy; SOC: solid organ cancer; SOT: Solid-organ transplantation; ONMD: other nonmalignant disease; EORTC/MSG: European Organization for Research and Treatment of Cancer/Invasive Fungal Infections Cooperative Group and the National Institute of Allergy and Infectious Diseases Mycoses Study Group; ① all-caused mortality at week 12; ② overall response rate; ③ adverse events; ④ severe adverse events; POS: posaconazole, 300 mg/day; TIS: Treatment with T-cell immunosuppressant drugs; CT: corticosteroid treatment; ISID: inherited severe immunodeficiency; MDS: myelodysplastic syndrome; ISA: isavuconazole, 200 mg/day; CLL: chronic lymphocytic leukaemia; AA: aplastic anaemia; CML: chronic myeloid leukaemia; MM: multiple myeloma; COPD: chronic obstructive pulmonary disease; HD: Hodgkin’s disease; DM: diabetes mellitus; LRTD: lower respiratory tract disease.

### 3.2 Risk of bias abias assessment

**FIGURE 3:**
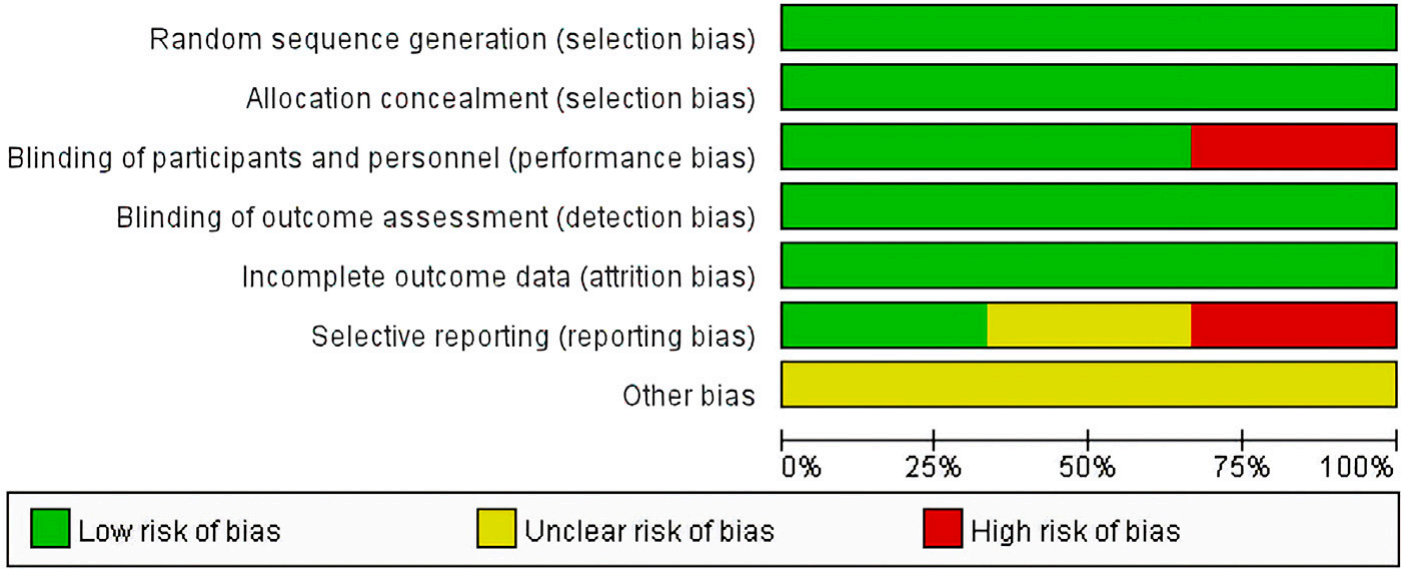
Risk of bias graph.

**FIGURE 4:**
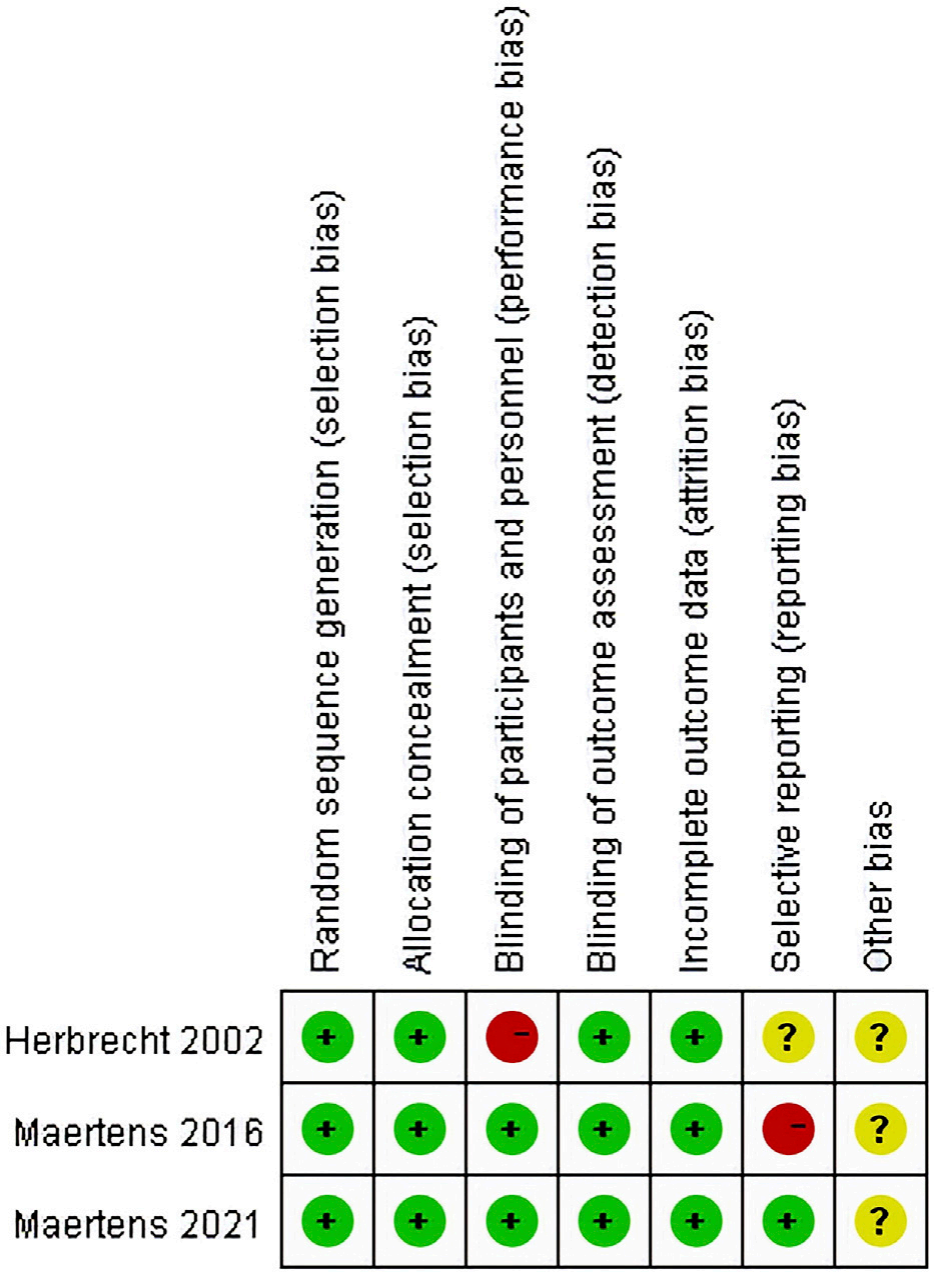
Risk of bias summary.

All studies (Herbrecht et al., 2015; Maertens et al., 2021; Maertens et al., 2016) had a low risk of selection bias in the randomization and allocation concealment after applying central randomization and a central interactive voice response system. Two studies (Maertens et al., 2021; Maertens et al., 2016) had a low risk of performance bias and detection bias, as both participants and study personnel were masked; however, this risk was high in the study by Herbrecht et al. for the open-label trial design. All studies had a low risk of attrition bias, as there was no participants excluded from any efficacy or safety analyses. The Herbrecht et al. (2015) study did not mention registration information, so it was unclear whether all the pre-designed outcomes had been reported. The study by Maertens et al. (2021) reported all predesigned outcomes. The Maertens 2016 study (Maertens et al., 2016) did not report SAEs that were predetermined in the protocol, so it was rated as a high risk of reporting bias. All three studies were supported by pharmaceutical companies but the role of sponsors in the research was not mentioned, so the risk of bias caused by conflict of interest was unclear (Figure 3; Figure 4).

### 3.3 Results of network meta-analysis

#### 3.3.1 All-cause mortality at week 12 for patients with proven and probable IA

All three RCTs reported the all-cause mortality at week 12 for patients with proven and probable IA. Results from the NMA (eFigure 1A in the Supplementary Material and Table 2) indicate that both VCZ and ISA significantly reduced the all-cause mortality at week 12 compared to D-AmB (VCZ vs D-AmB: *RR* = 0.66, *95%CI* = 0.47–0.93, moderate certainty; ISA vs D-AmB: *RR* = 0.52, *95%CI* = 0.31–0.86, low certainty), while the mortality of POS was similar to D-AmB (*RR* = 0.73, *95%CI* = 0.46–1.16, low certainty). The probability test shows that ISA was the best treatment (SUCRA = 93.50%; mean rank, 1.20; Table 3).

**TABLE 2:** Relative risk of various antifungals from the NMA for all outcomes.

Treatment	All-cause mortality at week 12 for patients with proven and probable IA	All-cause mortality at week 12 for patients with proven, probable and possible IA	Overall response rate	Adverse events	Severe adverse events
ISA vs VCZ	0.79 (0.54,1.15)^c^	0.94 (0.70,1.26)^c^	0.96 (0.70,1.32)^c^	0.97 (0.94,1.00)^c^	0.91 (0.77,1.06)^c^
ISA vs POS	0.71 (0.44,1.16)^c^	1.02 (0.68,1.53)^c^	1.05 (0.70,1.56)^c^	0.97 (0.93,1.01)^c^	0.88 (0.72,1.08)^c^
ISA vs D-AmB	0.52 (0.31,0.86)^c,^ ^e^	0.60 (0.39,0.94)^c^ ^,^ ^e^	1.87 (1.16,3.03)^c^ ^,^ ^e^	*NA*	0.50 (0.31,0.80)^c^ ^,^ ^e^
VCZ vs POS	0.90 (0.66,1.23)^c^	1.09 (0.82,1.45)^c^	1.09 (0.86,1.39)^c^	1.00 (0.97,1.03)^b^	0.97 (0.85,1.11)^c^
VCZ vs D-AmB	0.66 (0.47,0.93)^b,^ ^e^	0.64 (0.46,0.90)^b^ ^,^ ^e^	1.95 (1.36,2.79)^b^ ^,^ ^e^	*NA*	0.55 (0.35,0.86)^b^ ^,^ ^e^
POS vs D-AmB	0.73 (0.46,1.16)^c^	0.59 (0.38,0.91)^c^ ^,^ ^e^	1.79 (1.16,2.75)^c^ ^,^ ^e^	*NA*	0.57 (0.36,0.90)^c^ ^,^ ^e^

Each number is an relative rare (=row/column), and 95% confidence interval; NMA: network meta-analysis; IA: invasive aspergillosis; VCZ: voriconazole, 8 mg/kg/day; 400 mg/day; D-AmB: amphotericin B deoxycholate, 1.0–1.5 mg/kg/day; POS: posaconazole, 300 mg/day; ISA: isavuconazole, 200 mg/day.

^a^ high certainty.

^b^ moderate certainty.

^c^ low certainty.

^d^ very low certainty.

^e^ significant difference; *NA*: not applicable.

**TABLE 3:** Surface under the cumulative ranking area and mean rank for all outcomes.

Treatment	All-cause mortality at week 12 for patients with proven and probable IA		All-cause mortality at week 12 for patients with proven, probable and possible IA		Overall response rate		Adverse events		Severe adverse events	
	SUCRA (%)	Mean rank	SUCRA (%)	Mean rank	SUCRA (%)	Mean rank	SUCRA (%)	Mean rank	SUCRA (%)	Mean rank
D-AmB	3.30	3.90	1.00	4.00	0.30	4.00	*NA*	*NA*	0.40	4.00
VCZ	61.50	2.20	53.50	2.40	79.00	1.60	25.40	2.50	60.10	2.20
POS	41.60	2.80	74.70	1.80	55.00	2.30	28.20	2.40	47.00	2.60
ISA	93.50	1.20	70.90	1.90	65.60	2.00	96.30	1.10	92.50	1.20

IA: invasive aspergillosis; SUCRA: surface under the cumulative ranking area; D-AmB: amphotericin B deoxycholate, 1.0–1.5 mg/kg/day; VCZ: voriconazole, 8 mg/kg/day; 400 mg/day; POS: posaconazole, 300 mg/day; ISA: isavuconazole, 200 mg/day; *NA*: not applicable.

#### 3.3.2 All-cause mortality at week 12 for patients with proven, probable and possible IA

All three RCTs reported all-cause mortality at week 12 for patients with proven, probable and possible IA. In comparison to D-AmB, triazoles significantly reduced all-cause mortality at week 12. However, there was no significant difference among triazoles (eFigure 1B in the Supplementary Material and Table 2). The probability of each treatment being the most superior was shown in Table 3, which indicates that POS (SUCRA = 74.70%; mean rank, 1.80) was the best treatment.

#### 3.3.3 Overall response rate

The overall response rate was reported in all studies. In comparison to D-AmB, triazoles significantly improved the overall response rate. However, there was no significant difference among triazoles (eFigure 1C in the Supplementary Material and Table 2). The probability of each treatment being the most superior was shown in Table 3, which indicates that VCZ (SUCRA = 79.00%; mean rank, 1.60) was the best treatment.

#### 3.3.4 Overall AEs

Two RCTs (Maertens et al., 2021; Maertens et al., 2016) reported the overall AEs. As shown in eFigure 1D in the Supplementary Material and Table 2, the incidence of overall AEs among all the triazoles was not significantly different. The probability of each treatment being the most superior was shown in Table 3, which indicates that ISA (SUCRA = 96.30%; mean rank, 1.10) was the best treatment. The most common AEs included nausea, vomiting, diarrhea, pyrexia/chill, hypokalaemia, eye disorders, skin and subcutaneous tissue disorders, psychiatric disorders, investigations (abnormal laboratory tests), hepatobiliary disorders, metabolism, and nutrition disorders (Table 4).

**TABLE 4:** The incidence of adverse events for different antifungals.

Adverse events	D-AmB	ISA	POS	VCZ
Nausea (%)	*NA*	27.63	4.17	16.30
Vomiting (%)	*NA*	24.90	3.13	14.29
Diarrhoea (%)	*NA*	23.74	1.39	11.36
Pyrexia/Chill (%)	24.86	32.68	NR	23.62
Hypokalaemia (%)	*NA*	17.51	3.82	10.99
Eye disorders (%)	4.32	15.18	1.74	24.86
Skin and subcutaneous tissue disorders (%)	3.24	33.46	1.74	18.65
Psychiatric disorders (%)	2.7	27.24	2.08	16.35
Investigations (abnormal laboratory tests) (%)	*NA*	33.07	14.93	23.99
Hepatobiliary disorders (%)	*NA*	8.95	3.13	9.52
Metabolism and nutrition disorders (%)	*NA*	42.02	6.25	23.44

D-AmB: amphotericin B deoxycholate, ISA: isavuconazole, 200 mg/day; 1.0–1.5 mg/kg/day; POS: posaconazole, 300 mg/day; VCZ: voriconazole, 8 mg/kg/day; 400 mg/day; *NA*: not applicable.

#### 3.3.5 SAEs

Two RCTs (Maertens et al., 2021; Maertens et al., 2016) reported the SAEs. The Maertens 2016 study (Maertens et al., 2016) did not report SAEs in the full-text, so we extracted the data from its protocol. The incidence of SAEs for triazoles was significantly lower than D-AmB (eFigure 1E in the Supplementary Material and Table 2), though not significantly different among all three triazoles. The probability of each treatment being the most superior was shown in Table 3, which indicates that ISA (SUCRA = 92.50%; mean rank, 1.20) was the best treatment. The most common SAEs included renal and urinary disorders, hypokalaemia, metabolism and nutrition disorders, abnormal liver function, general disorders, dyspnea, gastrointestinal disorders, hypotension, blood and lymphatic system disorders, rash, nervous system disorders, and eye disorders (Table 5).

**TABLE 5:** The incidence of severe adverse events for different antifungals.

Severe adverse events	D-AmB	ISA	POS	VCZ
Renal and urinary disorders (%)	10.27	3.89	3.47	2.43
Hypokalaemia (%)	3.24	0	1.39	0.27
Metabolism and nutrition disorders (%)	1.08	0.78	3.13	1.89
Abnormal liver function (%)	2.16	0	1.04	1.35
General disorders (%)	3.78	5.45	4.17	4.86
Dyspnea (%)	22.16	1.95	0.35	0.14
Gastrointestinal disorders (%)	0	4.67	4.86	4.86
Hypotension (%)	0.54	0.39	1.04	0.41
Blood and lymphatic system disorders (%)	0	12.06	11.81	6.76
Rash (%)	0.54	0	0	0.68
Nervous system disorders (%)	0	7.00	4.51	5.68
Eye disorders (%)	0	0.78	0	0.54

D-AmB: amphotericin B deoxycholate, ISA: isavuconazole, 200 mg/day; 1.0–1.5 mg/kg/day; POS: posaconazole, 300 mg/day; VCZ: voriconazole, 8 mg/kg/day; 400 mg/day.

## 4 Discussion

This study performed an NMA to evaluate the efficacy and safety of different monotherapies in primary therapy of IA patients. We find that, compared to D-AmB, the triazoles (POS, ISA and VCZ) can improve the overall response rate but only VCZ and ISA can reduce the all-cause mortality at week 12 in patients with proven and probable IA. ISA seemed to be the most effective treatment in the above population. As to proven, probable, and possible IA patients, the triazoles were superior to D-AmB in terms of reducing all-cause mortality, with POS being the most effective treatment. Although the risk of SAEs for the triazoles was significantly lower than that for D-AmB, no significant difference existed among the triazoles in terms of SAEs or overall AEs.

Most guidelines (Patterson et al., 2016; Frederic et al., 2017; Ullmann et al., 2018; Garcia-Vidal et al., 2019; Douglas et al., 2021) recommended VCZ as the first-line therapy in primary therapy of IA. Nevertheless, this NMA found no significant difference in the efficacy among VCZ, ISA, and POS. As novel broad-spectrum triazoles with fungicidal activity against Aspergillus spp (Ostrosky-Zeichner et al., 2003; Sabatelli et al., 2006; Thompson and Wiederhold, 2010; Thompson et al., 2009), ISA and POS appeared to be superior treatments for proven/probable and proven/probable/possible IA patients in terms of reducing the all-cause mortality at week 12, respectively. A previous NMA (Liu et al., 2024) including 5 RCTs and 7 observational studies indicated that ISA was associated with the best probability of favorable response among the monotherapies for proven and probable IA patients, against our conclusions that VCZ was the best. The main reason could be, in the context of the NMA lacking direct comparison and closing loop, we only included RCTs rather than observational studies. In addition, the previous NMA applied the data from the modified ITT population (patients with proven or probable invasive mould disease) instead of the mycological ITT population (patients with proven or probable IA) into the final analyses. On the other hand, we excluded the RCT investigating different formulations of AmB because it failed to provide the head-to-head comparison with other triazoles but increased the uncertainty of the results of NMA. Furthermore, we also excluded the RCTs regarding the combination therapy because the guidelines (Frederic et al., 2017; Ullmann et al., 2018; Garcia-Vidal et al., 2019) did not recommend combination therapy as the first-line option in primary therapy of IA. Another multicenter retrospective study (Batista et al., 2023) indicated that ISA, VCZ or AmB had a comparable outcome in patients with underlying malignancy and a transplant, but the results should be interpreted with caution due to the small sample size and recall or reporting bias. Notably, Central nervous system (CNS) aspergillosis is a fatal disease with inevitable death if detected late (Patterson et al., 2016; Schwartz et al., 2007). Meena et al. (Meena et al., 2021) performed a systematic review of 235 cases with proven CNS aspergillosis and discovered that patients treated with VCZ were more likely to survive. Considering the excellent CNS penetration (∼50%), VCZ was the first choice for patients whose CNS involvement in IA was suspected (Patterson et al., 2016; Miceli, 2019). Another set of clinical data indicates that ISA shows satisfactory activity in IA located in CNS when compared to VCZ (Maertens et al., 2016; Schwartz et al., 2019). On the contrary, POS may not be the optimal treatment choice due to its limited ability to reach the cerebrospinal fluid and brain (Reinwald et al., 2009).

Results from our NMA indicate ISA is superior to other triazoles and D-AmB in terms of safety profile, which is consistent with the conclusion of a multicenter retrospective study (Batista et al., 2023). The significantly higher risk of SAEs, particularly nephrotoxicity, limits the use of AmB, so many guidelines (Patterson et al., 2016; Frederic et al., 2017; Ullmann et al., 2018; Garcia-Vidal et al., 2019) recommend it as an alternative or second-line therapy for patients who are intolerant or refractory to triazoles. A rapid vasoconstrictive effect of AmB on the afferent renal arterioles could cause a decrease in renal blood flow and a decrease in the glomerular filtration rate (Shirley and Scott, 2016), resulting in a further decline of renal function, especially in patients who already have acute kidney injury (Armstrong-James et al., 2020).

Several pharmacoeconomic studies have evaluated the cost-effectiveness of these first-line monotherapies in the primary therapy of IA. Some studies found that VCZ was dominant to AmB (Grau Cerrato et al., 2005; Jansen et al., 2006; Jansen et al., 2005; Rotstein et al., 2004; Wingard et al., 2007; Wenzel et al., 2005), while others found ISA was more cost-effective than VCZ (Azanza et al., 2021; Beauchemin et al., 2022; Floros et al., 2019; Harrington et al., 2017; Han et al., 2023) and POS was not economically advantageous over VCZ (Han et al., 2023) in the primary therapy of IA. Overall, ISA seemed to be the most advantageous among the above monotherapies based on current evidence.

In the retrieval strategy of this article, we also searched for echinocandins antifungals (such as micafungin, anidulafungin, and caspofungin), but finally, no study regarding echinocandins met the inclusion criteria. The reason was that echinocandins are predominantly applied for second-line or salvage therapy rather than primary therapy against IA (Patterson et al., 2016; Garcia-Vidal et al., 2019; Douglas et al., 2021). We also retrieved the first-generation antifungals such as fluconazole and itraconazole, but they were not employed for primary therapy of IA due to several clinically important limitations on their range of activity, the development of resistance, and some toxicity (Kale and Johnson, 2005). During literature screening, we excluded Kohno 2023 (Kohno et al., 2023) because few IA subjects were included. On the other hand, because the Herbrecht 2002 study (Herbrecht et al., 2002) only reported the number of overall AEs rather than the number of patients with overall AEs, we cannot compare the risk of overall AEs of D-AmB to triazoles.

There are several limitations in this study. First, we only included three RCTs in this systematic review, and the NMA was mainly based on the indirect comparison. Thus, the results should be verified by head-to-head RCTs with a large sample size and good methodological design. Second, the definitions for the overall response rate varied among different RCTs, which could introduce clinical heterogeneity into the NMA. Third, RCTs included in this NMA were not sensitive enough to identify rare AEs related to the medications as the sample size was relatively small. Fourth, due to a few trials reporting the results of patients with different underlying diseases, we did not perform the subgroup analyses. Considering the lower power of the test and heterogeneity caused by response definitions and patient population, the conclusions of this study should be interpreted with caution.

## 5 Conclusion

Although the efficacy and safety of VCZ, ISA, and POS are similar in the primary therapy of IA, ISA seems to be the optimal choice for patients with IA.

## Data Availability

The original contributions presented in the study are included in the article/Supplementary Material, further inquiries can be directed to the corresponding author.

## Abbreviations

IA, invasive aspergillosis; NMA, network meta-analysis; CBM, China Biology Medicine; RCTs, Randomized controlled trials; GRADE, Grading of Recommendations Assessment, Development and Evaluation; AEs, adverse events; SAEs, severe adverse events; D-AmB, amphotericin B deoxycholate; POS, posaconazole; ISA, isavuconazole; VCZ, voriconazole; IPA, invasive pulmonary aspergillosis; HSCT, hematopoietic stem cell transplantation; SOT, solid organ transplant; EORTC/MSG, European Organization for Research and Treatment of Cancer Mycoses Study Group; IDSA, Infectious Diseases Society of America; (ECIL)-6, European Conference on Infection in Leukemia; ESCMID-ECMM-ERS, European Society for Clinical Microbiology and Infectious Diseases-European Confederation of Medical Mycology-European Respiratory Society guideline; GEMICOMED-SEIMC/REIPI, Group of Fungal Infections from the Spanish Society of Infectious Diseases and Clinical Microbiology; FDA, Food and Drug Administration; RR, relative risk; CI, Confidence interval; SUCRA, Surface Under the Cumulative Ranking Area.
