# Supplementary material for: The efficacy and safety of first-line monotherapies in primary therapy of invasive aspergillosis: a systematic review
Source: Front Pharmacol. 2025 Jan 15;15:1530999. doi: 10.3389/fphar.2024.1530999 (PMC11775403; doi:10.3389/fphar.2024.1530999)
Supplement: Supplementary file 1 [file DataSheet1.docx]

Supplementary Material

# eTable 1. PRISMA NMA Checklist of Items to Include When Reporting A Systematic Review Involving a Network Meta-analysis

# eTable 2. Searching Strategy

# eTable 3. The criteria for proven, probable and possible IFD

# eTable 4. GRADE for all outcomes

# eFigure 1. Forest plots of network meta-analyses for outcomes of various antifungalsSupplementary Figures and Tables

**eTable 1. PRISMA NMA Checklist of Items to Include When Reporting A Systematic Review Involving a Network Meta-analysis**

| **Section/Topic** | **Item #** | **Checklist Item** | **Reported on Page #** |
| --- | --- | --- | --- |
| **TITLE** |  |  |  |
| Title | 1 | Identify the report as a systematic review *incorporating a network meta-analysis (or related form of meta-analysis).* | Page 1 |
|  |  |  |  |
| **ABSTRACT** |  |  |  |
| Structured summary | 2 | Provide a structured summary including, as applicable:  **Background:** main objectives  **Methods:** data sources; study eligibility criteria, participants, and interventions; study appraisal; and *synthesis methods, such as network meta-analysis.*  **Results:** number of studies and participants identified; summary estimates with corresponding confidence/credible intervals; *treatment rankings may also be discussed. Authors may choose to summarize pairwise comparisons against a chosen treatment included in their analyses for brevity.*  **Discussion/Conclusions:** limitations; conclusions and implications of findings.  **Other:** primary source of funding; systematic review registration number with registry name. | Page 1 to Page 2 |
|  |  |  |  |
| **INTRODUCTION** |  |  |  |
| Rationale | 3 | Describe the rationale for the review in the context of what is already known*, including mention of why a network meta-analysis has been conducted.* | Page 2 to Page 3 |
| Objectives | 4 | Provide an explicit statement of questions being addressed, with reference to participants, interventions, comparisons, outcomes, and study design (PICOS). | Page 3 to Page 4 |
|  |  |  |  |
| **METHODS** |  |  |  |
| Protocol and registration | 5 | Indicate whether a review protocol exists and if and where it can be accessed (e.g., Web address); and, if available, provide registration information, including registration number. | PROSPERO, CRD42023407632 |
| Eligibility criteria | 6 | Specify study characteristics (e.g., PICOS, length of follow-up) and report characteristics (e.g., years considered, language, publication status) used as criteria for eligibility, giving rationale. *Clearly describe eligible treatments included in the treatment network, and note whether any have been clustered or merged into the same node (with justification).* | Page 3 to Page 4 |
| Information sources | 7 | Describe all information sources (e.g., databases with dates of coverage, contact with study authors to identify additional studies) in the search and date last searched. | Page 4 |
| Search | 8 | Present full electronic search strategy for at least one database, including any limits used, such that it could be repeated. | eTable 2 |
| Study selection | 9 | State the process for selecting studies (i.e., screening, eligibility, included in systematic review, and, if applicable, included in the meta-analysis). | Page 4 |
| Data collection process | 10 | Describe method of data extraction from reports (e.g., piloted forms, independently, in duplicate) and any processes for obtaining and confirming data from investigators. | Page 4 |
| Data items | 11 | List and define all variables for which data were sought (e.g., PICOS, funding sources) and any assumptions and simplifications made. | Page 4 |
| **Geometry of the network** | **S1** | Describe methods used to explore the geometry of the treatment network under study and potential biases related to it. This should include how the evidence base has been graphically summarized for presentation, and what characteristics were compiled and used to describe the evidence base to readers. | Page 4 and Figure 2 |
| Risk of bias within individual studies | 12 | Describe methods used for assessing risk of bias of individual studies (including specification of whether this was done at the study or outcome level), and how this information is to be used in any data synthesis. | Page 4 |
| Summary measures | 13 | State the principal summary measures (e.g., risk ratio, difference in means). *Also describe the use of additional summary measures assessed, such as treatment rankings and surface under the cumulative ranking curve (SUCRA) values, as well as modified approaches used to present summary findings from meta-analyses.* | Page 5 |
| Planned methods of analysis | 14 | Describe the methods of handling data and combining results of studies for each network meta-analysis. This should include, but not be limited to:   - *Handling of multi-arm trials;* - *Selection of variance structure;* - *Selection of prior distributions in Bayesian analyses; and* - *Assessment of model fit.* | Page 5 |
| **Assessment of Inconsistency** | **S2** | Describe the statistical methods used to evaluate the agreement of direct and indirect evidence in the treatment network(s) studied. Describe efforts taken to address its presence when found. | Page 5 |
| Risk of bias across studies | 15 | Specify any assessment of risk of bias that may affect the cumulative evidence (e.g., publication bias, selective reporting within studies). | Page 5 |
| Additional analyses | 16 | Describe methods of additional analyses if done, indicating which were pre-specified. This may include, but not be limited to, the following:   - Sensitivity or subgroup analyses; - Meta-regression analyses; - *Alternative formulations of the treatment network; and* - *Use of alternative prior distributions for Bayesian analyses (if applicable).* | Page 5 |
|  |  |  |  |
| **RESULTS†** |  |  |  |
| Study selection | 17 | Give numbers of studies screened, assessed for eligibility, and included in the review, with reasons for exclusions at each stage, ideally with a flow diagram. | Page 5 and Figure 1 |
| **Presentation of network structure** | **S3** | Provide a network graph of the included studies to enable visualization of the geometry of the treatment network. | Figure 2 |
| **Summary of network geometry** | **S4** | Provide a brief overview of characteristics of the treatment network. This may include commentary on the abundance of trials and randomized patients for the different interventions and pairwise comparisons in the network, gaps of evidence in the treatment network, and potential biases reflected by the network structure. | Page 5 |
| Study characteristics | 18 | For each study, present characteristics for which data were extracted (e.g., study size, PICOS, follow-up period) and provide the citations. | Page 5 and Table 1 |
| Risk of bias within studies | 19 | Present data on risk of bias of each study and, if available, any outcome level assessment. | Page 6 and Figure 4 |
| Results of individual studies | 20 | For all outcomes considered (benefits or harms), present, for each study: 1) simple summary data for each intervention group, and 2) effect estimates and confidence intervals. *Modified approaches may be needed to deal with information from larger networks.* | Page 6 to Page 7 |
| Synthesis of results | 21 | Present results of each meta-analysis done, including confidence/credible intervals. *In larger networks, authors may focus on comparisons versus a particular comparator (e.g. placebo or standard care), with full findings presented in an appendix. League tables and forest plots may be considered to summarize pairwise comparisons.* If additional summary measures were explored (such as treatment rankings), these should also be presented. | Page 6 to Page 7.  Table 2 to Table 5 and eFigure 1a to eFigure 1e in the Supplement. |
| **Exploration for inconsistency** | **S5** | Describe results from investigations of inconsistency. This may include such information as measures of model fit to compare consistency and inconsistency models, *P* values from statistical tests, or summary of inconsistency estimates from different parts of the treatment network. | Page 6 to Page 7 |
| Risk of bias across studies | 22 | Present results of any assessment of risk of bias across studies for the evidence base being studied. | Page 6 |
| Results of additional analyses | 23 | Give results of additional analyses, if done (e.g., sensitivity or subgroup analyses, meta-regression analyses*, alternative network geometries studied, alternative choice of prior distributions for Bayesian analyses,* and so forth). | eTable 4 |
|  |  |  |  |
| **DISCUSSION** |  |  |  |
| Summary of evidence | 24 | Summarize the main findings, including the strength of evidence for each main outcome; consider their relevance to key groups (e.g., healthcare providers, users, and policy-makers). | Page 7 to Page 8 |
| Limitations | 25 | Discuss limitations at study and outcome level (e.g., risk of bias), and at review level (e.g., incomplete retrieval of identified research, reporting bias). *Comment on the validity of the assumptions, such as transitivity and consistency. Comment on any concerns regarding network geometry (e.g., avoidance of certain comparisons).* | Page 8 |
| Conclusions | 26 | Provide a general interpretation of the results in the context of other evidence, and implications for future research. | Page 8 |
|  |  |  |  |
| **FUNDING** |  |  |  |
| Funding | 27 | Describe sources of funding for the systematic review and other support (e.g., supply of data); role of funders for the systematic review. This should also include information regarding whether funding has been received from manufacturers of treatments in the network and/or whether some of the authors are content experts with professional conflicts of interest that could affect use of treatments in the network. | Page 9 |

PICOS = population, intervention, comparators, outcomes, study design.

* Text in italics indicateS wording specific to reporting of network meta-analyses that has been added to guidance from the PRISMA statement.

† Authors may wish to plan for use of appendices to present all relevant information in full detail for items in this section.

**eTable 2. Searching Strategy**

| **Database** | **Search Strategy** | **Results** |
| --- | --- | --- |
| PUBMED | #1 Aspergillus[MeSH Terms] OR aspergillus[All Fields]  #2 Aspergillosis[MeSH Terms] OR aspergillosis[All Fields]  #3 OR/#1-2  #4 “antifungal agents”[MeSH Terms]  #5 fluconazole[MeSH Terms] OR fluconazole[All Fields]  #6 voriconazole[MeSH Terms] OR voriconazole[All Fields]  #7 itraconazole[MeSH Terms] OR itraconazole[All Fields]  #8 posaconazole[All Fields]  #9 isavuconazole[All Fields]  #10 azoles[MeSH Terms] OR azole*[All Fields]  #11 triazoles[MeSH Terms] OR triazole*[All Fields]  #12 “amphotericin B”[MeSH Terms] OR “amphotericin B”[All Fields]  #13 echinocandins[MeSH Terms] OR echinocandin*[All Fields]  #14 micafungin[MeSH Terms] OR micafungin[All Fields]  #15 caspofungin[MeSH Terms] OR caspofungin[All Fields]  #16 anidulafungin[MeSH Terms] OR anidulafungin[All Fields]  #17 OR/#4-16  #18"Randomized Controlled Trials as Topic"[Mesh Terms] OR "Controlled Clinical Trial"[Publication Type]  #19 ((singl* OR doubl* OR trebl* OR tripl*[All Fields]) AND (blind* OR mask* [All Fields]))  #20 Placebo[MeSH Terms] OR placebo*[All Fields]  #21 randomly[All Fields] OR trial[All Fields]  #22 OR/#18-21  #23 #3 AND #17 AND #22 | 1,246 |
| EMBASE | #1 'Aspergillus'/exp OR aspergillus:ab,ti  #2 'aspergillosis'/exp OR aspergillosis:ab,ti  #3 OR/#1-2  #4 'antifungal agent'/exp  #5 'fluconazole'/exp OR fluconazole:ab,ti  #6 'voriconazole'/exp OR voriconazole:ab,ti  #7 'itraconazole'/exp OR itraconazole:ab,ti  #8 'posaconazole'/exp OR posaconazole:ab,ti  #9 'isavuconazole'/exp OR isavuconazole:ab,ti  #10 'pyrrole derivative'/exp OR azole*:ab,ti  #11 'triazole derivative'/exp OR triazole*:ab,ti  #12 'amphotericin B'/exp OR “amphotericin B”:ab,ti  #13 'echinocandin'/exp OR echinocandin*:ab,ti  #14 'micafungin'/exp OR micafungin:ab,ti  #15 'caspofungin'/exp OR caspofungin:ab,ti  #16 'anidulafungin'/exp OR anidulafungin:ab,ti  #17 OR/#4-16  #18 'randomized controlled trial'/exp OR 'controlled clinical trial'/exp  #19 (singl*:ab,ti OR doubl*:ab,ti OR trebl*:ab,ti OR tripl*:ab,ti) AND (blind*:ab,ti OR mask*:ab,ti)  #20 'Placebo'/exp OR placebo*:ab,ti  #21 randomly:ab,ti OR trial:ab,ti  #22 OR/#18-21  #23 #3 AND #17 AND #22 | 1,408 |
| COCHRANE | #1 Aspergillosis[MeSH] OR Aspergillus[MeSH]#2 aspergillus OR aspergillosis ti,ab,kw#3 #1 OR #2#4 fluconazole[MeSH]#5 voriconazole[MeSH]#6 itraconazole[MeSH]#7 azoles[MeSH]#8 triazoles[MeSH]#9 amphotericin B[MeSH]#10 echinocandins[MeSH Terms]#11 micafungin[MeSH Terms]#12 caspofungin[MeSH Terms]#13 anidulafungin[MeSH Terms]#14 Fluconazole OR vORiconazole OR itraconazole OR posaconazole OR isavuconazole OR azole* OR triazole* OR “amphotericin B” OR echinocandin* OR micafungin OR caspofungin OR anidulafungin#15 OR/#4-14#16 Randomized Controlled Trials as Topic[Mesh]#17 ((singl* OR doubl* OR trebl* OR tripl*) AND (blind* OR mask* ))#18 Placebos[MeSH ]#19 placebo*#20randomly OR trial#21 OR/#16-20#22 #3 AND #15 AND #21 | 359 |
| WOS | #1 (TS=(Aspergillus OR Aspergillosis) OR AB=(Aspergillus OR Aspergillosis)) AND (TS=(“antifungal agents” OR fluconazole OR voriconazole OR itraconazole OR azoles OR triazoles OR “amphotericin B” OR echinocandins OR micafungin OR caspofungin OR anidulafungin) OR AB=(“antifungal agents” OR fluconazole OR vORiconazole OR itraconazole OR posaconazole OR isavuconazole OR azole* OR triazole* OR “amphotericin B” OR echinocandin* OR micafungin OR caspofungin OR anidulafungin))#2 TS=("Randomized Controlled Trials as Topic" OR "randomized controlled trial" OR "Controlled Clinical Trial")#3 AB=((singl* OR doubl* OR trebl* OR tripl*) AND (blind* OR mask* ))#4 TS=(Placebo) OR AB=(placebo*)#5 AB=(randomly OR trial)#6 OR/#2-5#7 #1 AND #6 | 1,518 |

**eTable 3. The criteria for proven, probable and possible IFD**

| **Criteria for proven invasive fungal disease** |  |
| --- | --- |
| **Analysis and specimen** | **Mold^a^** |
| Microscopic analysis: sterile material | Histopathologic, cytopathologic, or direct microscopic examination^b^ of a specimen obtained by needle aspiration or biopsy in which hyphae or melanized yeast-like forms are seen accompanied by evidence of associated tissue damage |
| Culture: Sterile material | Recovery of a mold or “black yeast” by culture of a specimen obtained by a sterile procedure from a normally sterile and clinically or radiologically abnormal site consistent with an infectious disease process, excluding bronchoalveolar lavage fluid, a cranial sinus cavity specimen, and urine |
| Blood | Blood culture that yields a mold^c^ (e.g., Fusarium species) in the context of a compatible infectious disease process |
| Serological analysis: CSF | Not applicable |
| **Criteria for probable invasive fungal disease** |  |
| Host factors^d^ | Recent history of neutropenia (<0.5 × 109 neutrophils/L [<500 neutrophils/mm3 ] for >10 days) temporally related to the onset of fungal disease |
|  | Receipt of an allogeneic stem cell transplant |
|  | Prolonged use of corticosteroids (excluding among patients with allergic bronchopulmonary aspergillosis) at a mean minimum dose of 0.3 mg/kg/day of prednisone equivalent for >3 weeks |
|  | Treatment with other recognized T cell immunosuppressants, such as cyclosporine, TNF-α blockers, specific monoclonal antibodies (such as alemtuzumab), or nucleoside analogues during the past 90 days |
|  | Inherited severe immunodeficiency (such as chronic granulomatous disease or severe combined immunodeficiency) |
| Clinical criteria^e^ | Lower respiratory tract fungal disease^f^  The presence of 1 of the following 3 signs on CT: Dense, well-circumscribed lesions(s) with or without a halo sign /Air-crescent sign/Cavity |
|  | Tracheobronchitis  Tracheobronchial ulceration, nodule, pseudomembrane, plaque, or eschar seen on bronchoscopic analysis |
|  | Sinonasal infection    Imaging showing sinusitis plus at least 1 of the following 3 signs: Acute localized pain (including pain radiating to the eye)/Nasal ulcer with black eschar /Extension from the paranasal sinus across bony barriers, including into the orbit |
|  | CNS infection    1 of the following 2 signs: Focal lesions on imaging /Meningeal enhancement on MRI or CT |
| Mycological criteria | Direct test (cytology, direct microscopy, or culture)  Mold in sputum, bronchoalveolar lavage fluid, bronchial brush, or sinus aspirate samples, indicated by 1 of the following: Presence of fungal elements indicating a mold/Recovery by culture of a mold (e.g., Aspergillus, Fusarium, Zygomycetes, or Scedosporium species) |
|  | Indirect tests (detection of antigen or cell-wall constituents)^g^    Aspergillosis:Galactomannan antigen detected in plasma, serum, bronchoalveolar lavage fluid, or CSF    Invasive fungal disease other than cryptococcosis and zygomycoses:β-D-glucan detected in serum |

****NOTE.**** Probable IFD requires the presence of a host factor, a clinical criterion, and a mycological criterion. Cases that meet the criteria for a host factor and a clinical criterion but for which mycological criteria are absent are considered possible IFD.

^a^If culture is available, append the identification at the genus or species level from the culture results.

^b^Tissue and cells submitted for histopathologic or cytopathologic studies should be stained by Grocott-Gomorri methenamine silver stain or by periodic acid Schiff stain, to facilitate inspection of fungal structures. Whenever possible, wet mounts of specimens from foci related to invasive fungal disease should be stained with a fluorescent dye (e.g., calcofluor or blankophor).

^c^Recovery of Aspergillus species from blood cultures invariably represents contamination.

^d^Host factors are not synonymous with risk factors and are characteristics by which individuals predisposed to invasive fungal diseases can be recognized. They are intended primarily to apply to patients given treatment for malignant disease and to recipients of allogeneic hematopoietic stem cell and solidorgan transplants. These host factors are also applicable to patients who receive corticosteroids and other T cell suppressants as well as to patients with primary immunodeficiencies.

^e^Must be consistent with the mycological findings, if any, and must be temporally related to current episode.

^f^Every reasonable attempt should be made to exclude an alternative etiology. disseminated disease.

^g^These tests are primarily applicable to aspergillosis and candidiasis and are not useful in diagnosing infections due to Cryptococcus species or Zygomycetes (e.g., Rhizopus, Mucor, or Absidia species). Detection of nucleic acid is not included, because there are as yet no validated or standardized methods.

**eTable 4. GRADE for all outcomes**

Criteria for downgrading evidence items^1^

**Risk of bias:** We categorized the overall risk of bias for each study according to the Cochrane Risk of Bias tool. If one or more studies were unclear or high risk of bias, the item was rated as "some concern", otherwise it was rated as "no concern".

**Imprecision:** We considered a clinically meaningful threshold for SMD to be 0.50 and graded the degree of concerns according to the possible changes in clinical inference according to the following criteria:^2,3^


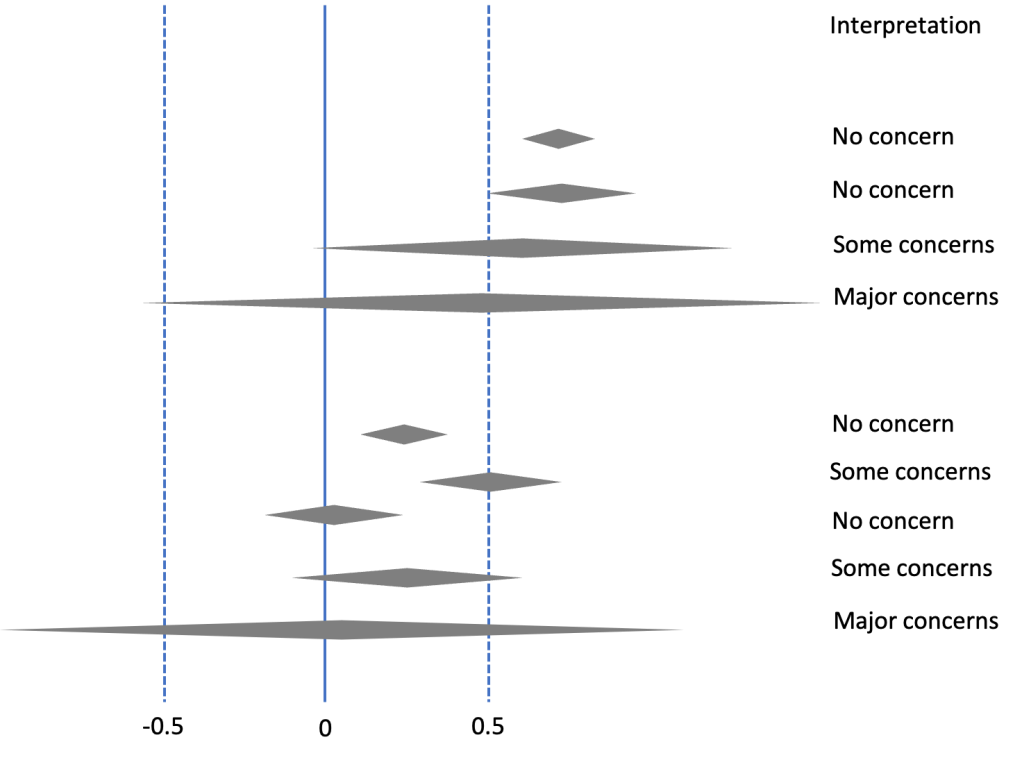


**Publication bias:** When there were less than 10 studies, no publication bias was detected and it was rated as "no concern"; when there were more than 10 studies were conducted, publication bias was detected using the egger test, the item was rated as "some concern" when the P＜0.05 and "no concern" when P>0.05.

**Incoherence:** Incoherence of direct and indirect evidence was assessed by net split method. We rated "no concerns" when the P>0.05 and "some concerns"when the P＜0.05.

**Indirectness and intransitivity:** According to the results of the network meta-regression, indirectness and intransitivity were rated as "no concerns" when P > 0.05; they were rated as "some concerns" when P < 0.05.

**Inconsistency:** We evaluated the degree of concerns through comparing the clinical inference based on the 95% confidence intervals (CI) and that based on the 95% prediction interval (PrI), the latter reflecting the degree of heterogeneity. Appling the same clinical inference framework as for imprecision, we saw "no concerns" in heterogeneity when the two judgements matched (e.g. no concern based on 95% CI and no concern based on 95% PrI), "some concerns" when they differed by one degree (e.g. no concern based on 95% CI but some concerns based on 95% PrI), and "major concerns" when they differed by two degrees (e.g. no concern based on 95% CI but major concerns based on 95% PrI).^3^

Certainty of evidence and definitions^4^

High certainty—We are very confident that the true effect lies close to that of the estimate of the effect.

Moderate certainty—We are moderately confident in the effect estimate. The true effect is likely to be close to the estimate of the effect, but there is a possibility that it is substantially different.

Low certainty—Our confidence in the effect estimate is limited. The true effect may be substantially different from the estimate of the effect.

Very low certainty—We have very little confidence in the effect estimate. The true effect is likely to be substantially different from the estimate of effect.

1.Salanti G, Del Giovane C, Chaimani A, Caldwell DM, Higgins JP. Evaluating the quality of evidence from a network meta-analysis. PLoS One. 2014;9(7):e99682. Published 2014 Jul 3. doi:10.1371/journal.pone.0099682

2.Edinger JD, Arnedt JT, Bertisch SM, et al. Behavioral and psychological treatments for chronic insomnia disorder in adults: an American Academy of Sleep Medicine systematic review, meta-analysis, and GRADE assessment. J Clin Sleep Med. 2021;17(2):263-298. doi:10.5664/jcsm.8988

3.Nikolakopoulou A, Higgins JPT, Papakonstantinou T, et al. CINeMA: An approach for assessing confidence in the results of a network meta-analysis. PLoS Med. 2020;17(4):e1003082. Published 2020 Apr 3. doi:10.1371/journal.pmed.1003082

4.Balshem H, Helfand M, Schünemann HJ, et al. GRADE guidelines:3. Rating the quality of evidence. J Clin Epidemiol 2011;64:401-6. doi:10.1016/j.jclinepi.2010.07.015

**GRADE for all-cause mortality at week 12 for patients with proven and probable invasive aspergillosis**

| **Comparisons groups** | | **Direct Evidence** | | | | | | | | | | | | | |
| --- | --- | --- | --- | --- | --- | --- | --- | --- | --- | --- | --- | --- | --- | --- | --- |
| **Arm 1** | **Arm 2 (Ref)** | **No. of study** | **No. of patients** | **I-square, %** | **MD (95%CI)** | **Egger’s value** | **RoB** | **Inconsistency** | **Indirectness** | **Publication bias** | **Direct rating without imprecision** | **Imprecision** | **Direct rating with imprecision** | **Direct is more precise than indirect?** | **Need to rate indirect?** |
| Amphotericin B deoxycholate | Voriconazole | 1 | 237 | 0% | 1.51 (1.08,2.12) | - | Some concerns | No concerns | No concerns | No concerns | Moderate | No concerns | Moderate | NA | NA |
| Posaconazole | Voriconazole | 1 | 334 | - | 1.11 (0.81,1.51) | - | Some concerns | No concerns | No concerns | No concerns | Moderate | No concerns | Moderate | NA | NA |
| Isavuconazole | Voriconazole | 1 | 231 | - | 0.79 (0.54,1.15) | - | Some concerns | No concerns | No concerns | No concerns | Moderate | No concerns | Moderate | NA | NA |
| Posaconazole | Amphotericin B deoxycholate |  |  |  |  |  |  |  |  |  |  |  |  |  |  |
| Isavuconazole | Amphotericin B deoxycholate |  |  |  |  |  |  |  |  |  |  |  |  |  |  |
| Posaconazole | Isavuconazole |  |  |  |  |  |  |  |  |  |  |  |  |  |  |

| **Indirect Evidence** | | | | | | |  | **Network Evidence** | | | | | |
| --- | --- | --- | --- | --- | --- | --- | --- | --- | --- | --- | --- | --- | --- |
| **RR (95%CI)** | **First order loop of the most contribution** | **Lowest of C1 and C2** | **Intransitivity** | **Indirect rating without imprecision** | **Imprecision** | **Indirect rating with imprecision** |  | **RR (95%CI)** | **Incoherence, P-value** | **Higher rating of direct and indirect without imprecision** | **Incoherence** | **NMA Imprecision** | **Final network rating** |
| 0.03 (-63.35, 63.41) |  |  |  |  |  |  |  | 1.51 (1.08,2.12) | 0.99 | Moderate | No concerns | No concerns | Moderate |
| -0.83 (-1.14, -0.51) |  |  |  |  |  |  |  | 1.11(0.81,1.51) | - | Moderate | Some concerns | No concerns | Low |
| -0.83 (-1.20, -0.45) |  |  |  |  |  |  |  | 0.79(0.54,1.15) | - | Moderate | Some concerns | No concerns | Low |
| 0.73 (0.46,1.16) | Voriconazole | Moderate | No concerns | Moderate | No concerns | Moderate |  | 0.73(0.46,1.16) | - | Moderate | Some concerns | No concerns | Low |
| 0.52 (0.31,0.86) | Voriconazole | Moderate | No concerns | Moderate | Some concerns | Moderate |  | 0.52(0.31,0.86) | - | Moderate | Some concerns | No concerns | Low |
| 1.41 (0.86,2.29) | Voriconazole | Moderate | No concerns | Moderate | Some concerns | Moderate |  | 1.41 (0.86,2.29) | - | Moderate | Some concerns | No concerns | Low |

**GRADE for all-cause mortality at week 12 for patients with proven, probable and possible invasive aspergillosis**

| **Comparisons groups** | | **Direct Evidence** | | | | | | | | | | | | | |
| --- | --- | --- | --- | --- | --- | --- | --- | --- | --- | --- | --- | --- | --- | --- | --- |
| **Arm 1** | **Arm 2 (Ref)** | **No. of study** | **No. of patients** | **I-square, %** | **MD (95%CI)** | **Egger’s value** | **RoB** | **Inconsistency** | **Indirectness** | **Publication bias** | **Direct rating without imprecision** | **Imprecision** | **Direct rating with imprecision** | **Direct is more precise than indirect?** | **Need to rate indirect?** |
| Amphotericin B deoxycholate | Voriconazole | 1 | 343 | 0% | 1.56(1.14,2.11) | - | Some concerns | No concerns | No concerns | No concerns | Moderate | No concerns | Moderate | NA | NA |
| Posaconazole | Voriconazole | 1 | 575 | - | 0.92(0.71,1.18) | - | Some concerns | No concerns | No concerns | No concerns | Moderate | No concerns | Moderate | NA | NA |
| Isavuconazole | Voriconazole | 1 | 516 | - | 0.94( 0.72,1.22) | - | Some concerns | No concerns | No concerns | No concerns | Moderate | No concerns | Moderate | NA | NA |
| Posaconazole | Amphotericin B deoxycholate |  |  |  |  |  |  |  |  |  |  |  |  |  |  |
| Isavuconazole | Amphotericin B deoxycholate |  |  |  |  |  |  |  |  |  |  |  |  |  |  |
| Posaconazole | Isavuconazole |  |  |  |  |  |  |  |  |  |  |  |  |  |  |

| **Indirect Evidence** | | | | | | |  | **Network Evidence** | | | | | |
| --- | --- | --- | --- | --- | --- | --- | --- | --- | --- | --- | --- | --- | --- |
| **RR (95%CI)** | **First order loop of the most contribution** | **Lowest of C1 and C2** | **Intransitivity** | **Indirect rating without imprecision** | **Imprecision** | **Indirect rating with imprecision** |  | **RR (95%CI)** | **Incoherence, P-value** | **Higher rating of direct and indirect without imprecision** | **Incoherence** | **NMA Imprecision** | **Final network rating** |
| 0.04 (-67.42, 67.50) |  |  |  |  |  |  |  | 1.56 (1.11,2.17) | 0.99 | Moderate | No concerns | No concerns | Moderate |
| -0.88 (-1.14, -0.63) |  |  |  |  |  |  |  | 0.92 (0.69,1.22) | - | Moderate | Some concerns | No concerns | Low |
| -0.88 (-1.15, -0.62) |  |  |  |  |  |  |  | 0.94 (0.70,1.26) | - | Moderate | Some concerns | No concerns | Low |
| 0.59 (0.38,0.91) | Voriconazole | Moderate | No concerns | Moderate | No concerns | Moderate |  | 0.59 (0.38,0.91) | - | Moderate | Some concerns | No concerns | Low |
| 0.60 (0.39,0.94) | Voriconazole | Moderate | No concerns | Moderate | Some concerns | Moderate |  | 0.60 (0.39,0.94) | - | Moderate | Some concerns | No concerns | Low |
| 0.98 (0.65,1.47) | Voriconazole | Moderate | No concerns | Moderate | Some concerns | Moderate |  | 0.98 (0.65,1.47) | - | Moderate | Some concerns | No concerns | Low |

**GRADE for overall response rate**

| **Comparisons groups** | | **Direct Evidence** | | | | | | | | | | | | | |
| --- | --- | --- | --- | --- | --- | --- | --- | --- | --- | --- | --- | --- | --- | --- | --- |
| **Arm 1** | **Arm 2 (Ref)** | **No. of study** | **No. of patients** | **I-square, %** | **MD (95%CI)** | **Egger’s value** | **RoB** | **Inconsistency** | **Indirectness** | **Publication bias** | **Direct rating without imprecision** | **Imprecision** | **Direct rating with imprecision** | **Direct is more precise than indirect?** | **Need to rate indirect?** |
| Amphotericin B deoxycholate | Voriconazole | 1 | 237 | 0% | 0.51(0.36,0.74) | - | Some concerns | No concerns | No concerns | No concerns | Moderate | No concerns | Moderate | NA | NA |
| Posaconazole | Voriconazole | 1 | 334 | - | 0.92(0.72,1.17) | - | Some concerns | No concerns | No concerns | No concerns | Moderate | No concerns | Moderate | NA | NA |
| Isavuconazole | Voriconazole | 1 | 272 | - | 0.96(0.70,1.32) | - | Some concerns | No concerns | No concerns | No concerns | Moderate | No concerns | Moderate | NA | NA |
| Posaconazole | Amphotericin B deoxycholate |  |  |  |  |  |  |  |  |  |  |  |  |  |  |
| Isavuconazole | Amphotericin B deoxycholate |  |  |  |  |  |  |  |  |  |  |  |  |  |  |
| Posaconazole | Isavuconazole |  |  |  |  |  |  |  |  |  |  |  |  |  |  |

| **Indirect Evidence** | | | | | | |  | **Network Evidence** | | | | | |
| --- | --- | --- | --- | --- | --- | --- | --- | --- | --- | --- | --- | --- | --- |
| **RR (95%CI)** | **First order loop of the most contribution** | **Lowest of C1 and C2** | **Intransitivity** | **Indirect rating without imprecision** | **Imprecision** | **Indirect rating with imprecision** |  | **RR (95%CI)** | **Incoherence, P-value** | **Higher rating of direct and indirect without imprecision** | **Incoherence** | **NMA Imprecision** | **Final network rating** |
| 0.67 (0.31, 1.03) |  |  |  |  |  |  |  | 0.51 (0.36,0.74) | 0.99 | Moderate | No concerns | No concerns | Moderate |
| 1.34 (1.10, 1.58) |  |  |  |  |  |  |  | 0.92 (0.72,1.17) | - | Moderate | Some concerns | No concerns | Low |
| 1.33(1.01, 1.65) |  |  |  |  |  |  |  | 0.96 (0.70,1.32) | - | Moderate | Some concerns | No concerns | Low |
| 1.79 (1.16,2.75) | Voriconazole | Moderate | No concerns | Moderate | No concerns | Moderate |  | 1.79 (1.16,2.75) | - | Moderate | Some concerns | No concerns | Low |
| 1.87 (1.16,3.03) | Voriconazole | Moderate | No concerns | Moderate | No concerns | Moderate |  | 1.87 (1.16,3.03) | - | Moderate | Some concerns | No concerns | Low |
| 0.95 (0.64,1.42) | Voriconazole | Moderate | No concerns | Moderate | No concerns | Moderate |  | 0.95 (0.64,1.42) | - | Moderate | Some concerns | No concerns | Low |

**GRADE for AEs**

| **Comparisons groups** | | **Direct Evidence** | | | | | | | | | | | | | |
| --- | --- | --- | --- | --- | --- | --- | --- | --- | --- | --- | --- | --- | --- | --- | --- |
| **Arm 1** | **Arm 2 (Ref)** | **No. of study** | **No. of patients** | **I-square, %** | **MD (95%CI)** | **Egger’s value** | **RoB** | **Inconsistency** | **Indirectness** | **Publication bias** | **Direct rating without imprecision** | **Imprecision** | **Direct rating with imprecision** | **Direct is more precise than indirect?** | **Need to rate indirect?** |
| Posaconazole | Voriconazole | 1 | 575 | 0.00% | 1.01(0.98,1.05) | - | Some concerns | No concerns | No concerns | No concerns | Moderate | No concerns | Moderate | NA | NA |
| Isavuconazole | Voriconazole | 1 | 516 | - | 0.97(0.94, 1.00) | - | Some concerns | No concerns | No concerns | No concerns | Moderate | No concerns | Moderate | NA | NA |
| Posaconazole | Isavuconazole |  |  |  |  |  |  |  |  |  |  |  |  |  |  |

| **Indirect Evidence** | | | | | | |  | **Network Evidence** | | | | | |
| --- | --- | --- | --- | --- | --- | --- | --- | --- | --- | --- | --- | --- | --- |
| **RR (95%CI)** | **First order loop of the most contribution** | **Lowest of C1 and C2** | **Intransitivity** | **Indirect rating without imprecision** | **Imprecision** | **Indirect rating with imprecision** |  | **RR (95%CI)** | **Incoherence, P-value** | **Higher rating of direct and indirect without imprecision** | **Incoherence** | **NMA Imprecision** | **Final network rating** |
|  |  |  |  |  |  |  |  | 1.00 (0.97,1.03) | 0.99 | Moderate | No concerns | No concerns | Moderate |
|  |  |  |  |  |  |  |  | 0.97 (0.94,1.00) | - | Moderate | Some concerns | No concerns | Low |
| 1.03 (0.99,1.07) | Voriconazole | Moderate | No concerns | Moderate | No concerns | Moderate |  | 1.03 (0.99,1.07) | - | Moderate | Some concerns | No concerns | Low |

**GRADE for SAEs**

| **Comparisons groups** | | **Direct Evidence** | | | | | | | | | | | | | |
| --- | --- | --- | --- | --- | --- | --- | --- | --- | --- | --- | --- | --- | --- | --- | --- |
| **Arm 1** | **Arm 2 (Ref)** | **No. of study** | **No. of patients** | **I-square, %** | **MD (95%CI)** | **Egger’s value** | **RoB** | **Inconsistency** | **Indirectness** | **Publication bias** | **Direct rating without imprecision** | **Imprecision** | **Direct rating with imprecision** | **Direct is more precise than indirect?** | **Need to rate indirect?** |
| Amphotericin B deoxycholate | Voriconazole | 1 | 379 | 0% | 1.81(1.17,2.81) | - | Some concerns | No concerns | No concerns | No concerns | Moderate | No concerns | Moderate | NA | NA |
| Posaconazole | Voriconazole | 1 | 575 | - | 1.03(0.90,1.18) | - | Some concerns | No concerns | No concerns | No concerns | Moderate | No concerns | Moderate | NA | NA |
| Isavuconazole | Voriconazole | 1 | 516 | - | 0.90(0.77,1.05) | - | Some concerns | No concerns | No concerns | No concerns | Moderate | No concerns | Moderate | NA | NA |
| Posaconazole | Amphotericin B deoxycholate |  |  |  |  |  |  |  |  |  |  |  |  |  |  |
| Isavuconazole | Amphotericin B deoxycholate |  |  |  |  |  |  |  |  |  |  |  |  |  |  |
| Posaconazole | Isavuconazole |  |  |  |  |  |  |  |  |  |  |  |  |  |  |

| **Indirect Evidence** | | | | | | |  | **Network Evidence** | | | | | |
| --- | --- | --- | --- | --- | --- | --- | --- | --- | --- | --- | --- | --- | --- |
| **RR (95%CI)** | **First order loop of the most contribution** | **Lowest of C1 and C2** | **Intransitivity** | **Indirect rating without imprecision** | **Imprecision** | **Indirect rating with imprecision** |  | **RR (95%CI)** | **Incoherence, P-value** | **Higher rating of direct and indirect without imprecision** | **Incoherence** | **NMA Imprecision** | **Final network rating** |
| 0.01(-37.24, 37.26) |  |  |  |  |  |  |  | 1.81 (1.17,2.82) | 0.99 | Moderate | No concerns | No concerns | Moderate |
| -1.12 (-1.26, -0.99) |  |  |  |  |  |  |  | 1.03 (0.90,1.18) | - | Moderate | Some concerns | No concerns | Low |
| -1.20 (-1.36, -1.05) |  |  |  |  |  |  |  | 0.91 (0.77,1.06) | - | Moderate | Some concerns | No concerns | Low |
| 0.57 (0.36,0.90) | Voriconazole | Moderate | No concerns | Moderate | No concerns | Moderate |  | 0.57 (0.36,0.90) | - | Moderate | Some concerns | No concerns | Low |
| 0.50 (0.31,0.80) | Voriconazole | Moderate | No concerns | Moderate | Some concerns | Moderate |  | 0.50 (0.31,0.80) | - | Moderate | Some concerns | No concerns | Low |
| 1.14 (0.93,1.40) | Voriconazole | Moderate | No concerns | Moderate | Some concerns | Moderate |  | 1.14 (0.93,1.40) | - | Moderate | Some concerns | No concerns | Low |

**eFigure 1 Forest plots of network meta-analyses for outcomes of various antifungals.**

**A.all-cause mortality at week 12 for patients with proven and probable invasive aspergillosis**


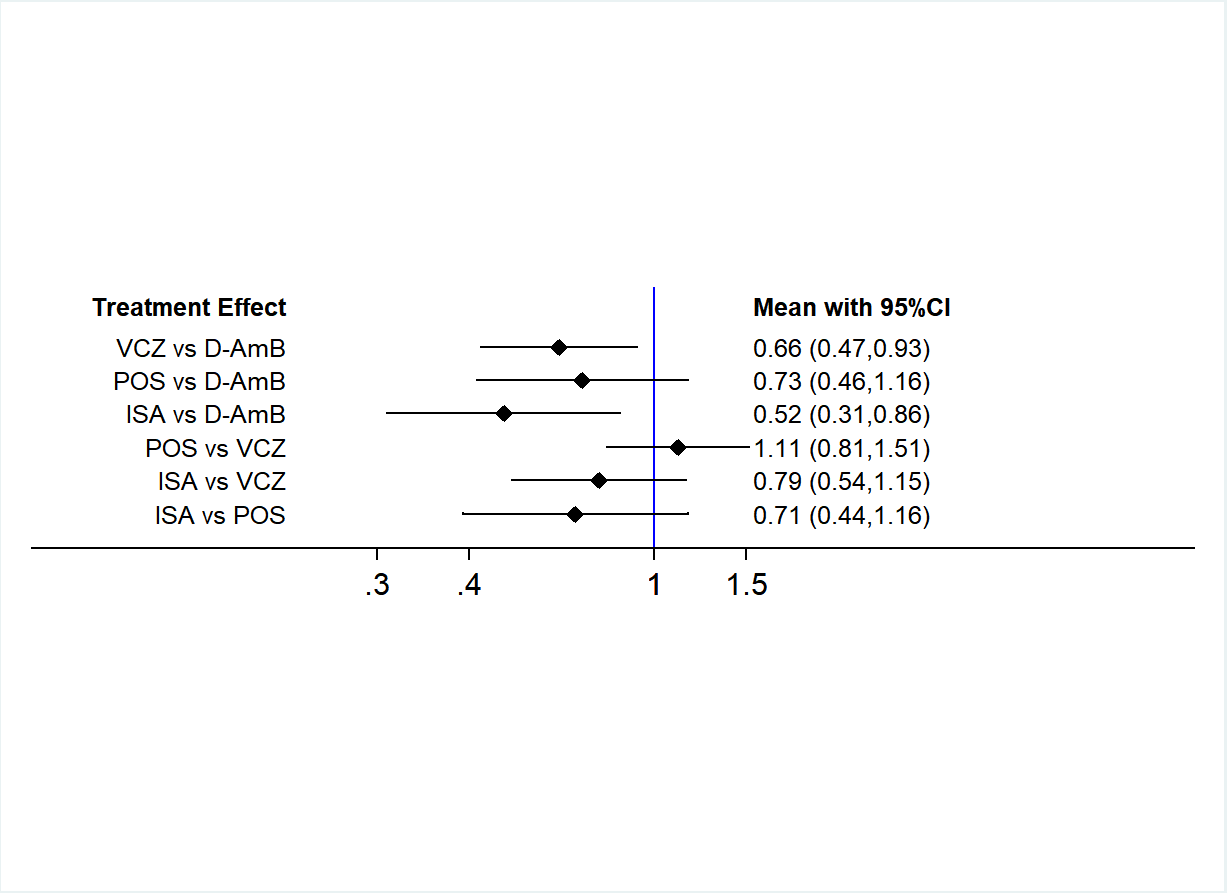


**B.all-cause mortality at week 12 for patients with proven, probable and possible invasive aspergillosis**


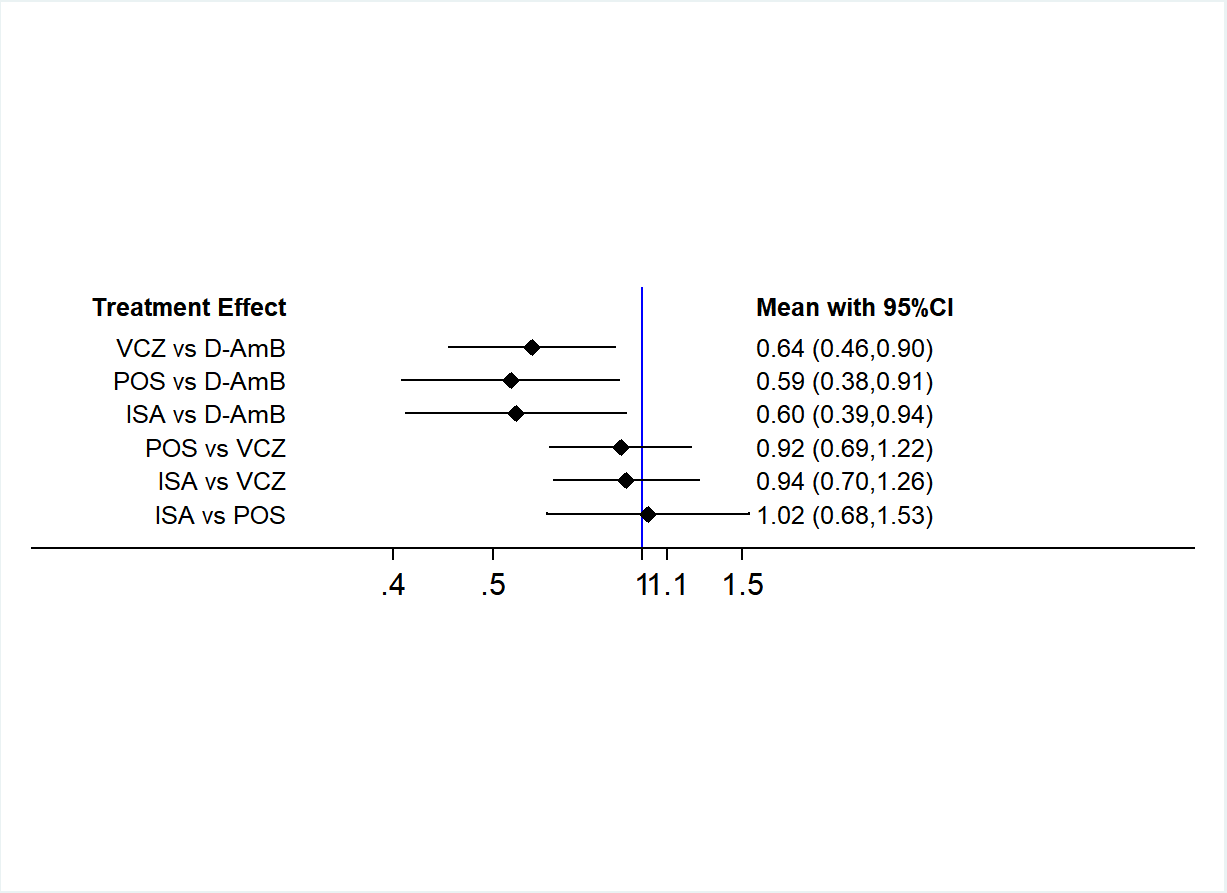


**C.overall response rate**


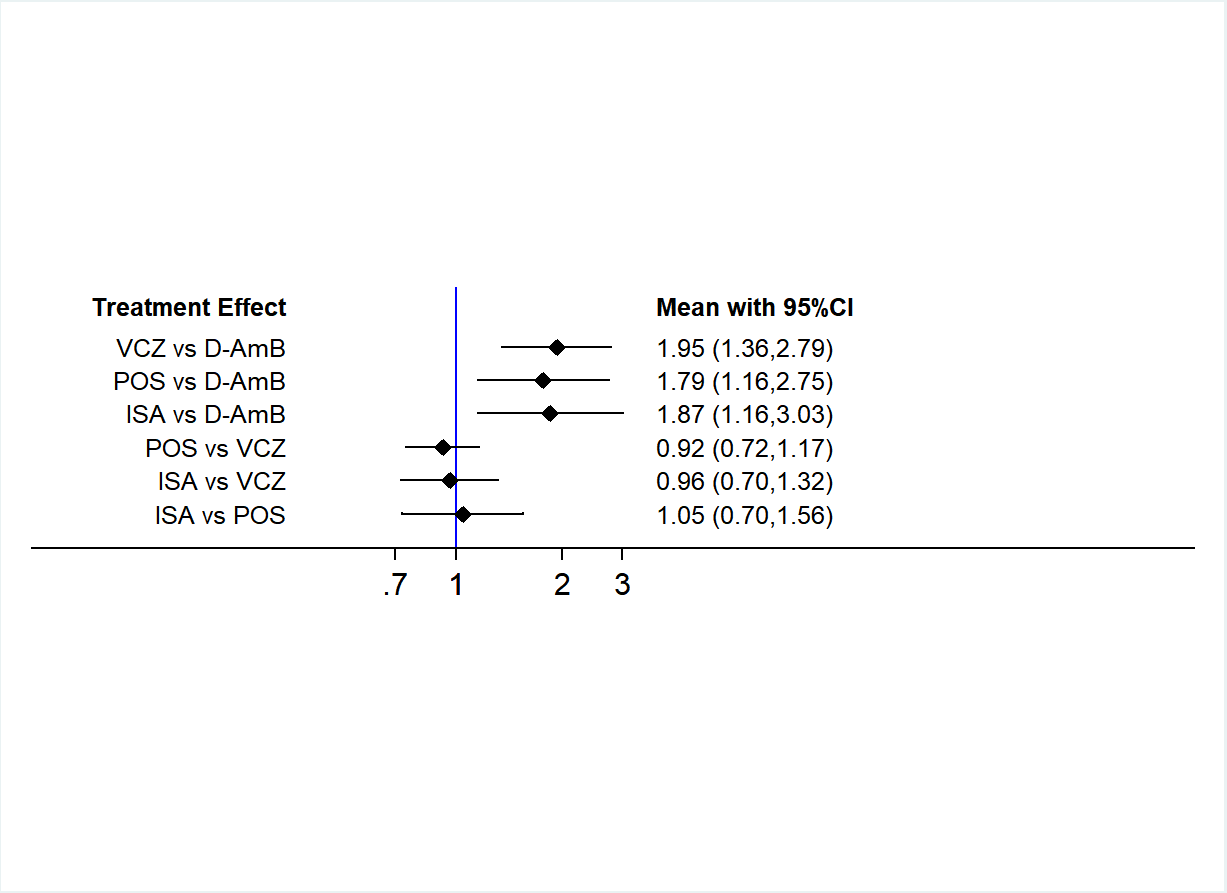


**D.adverse events**


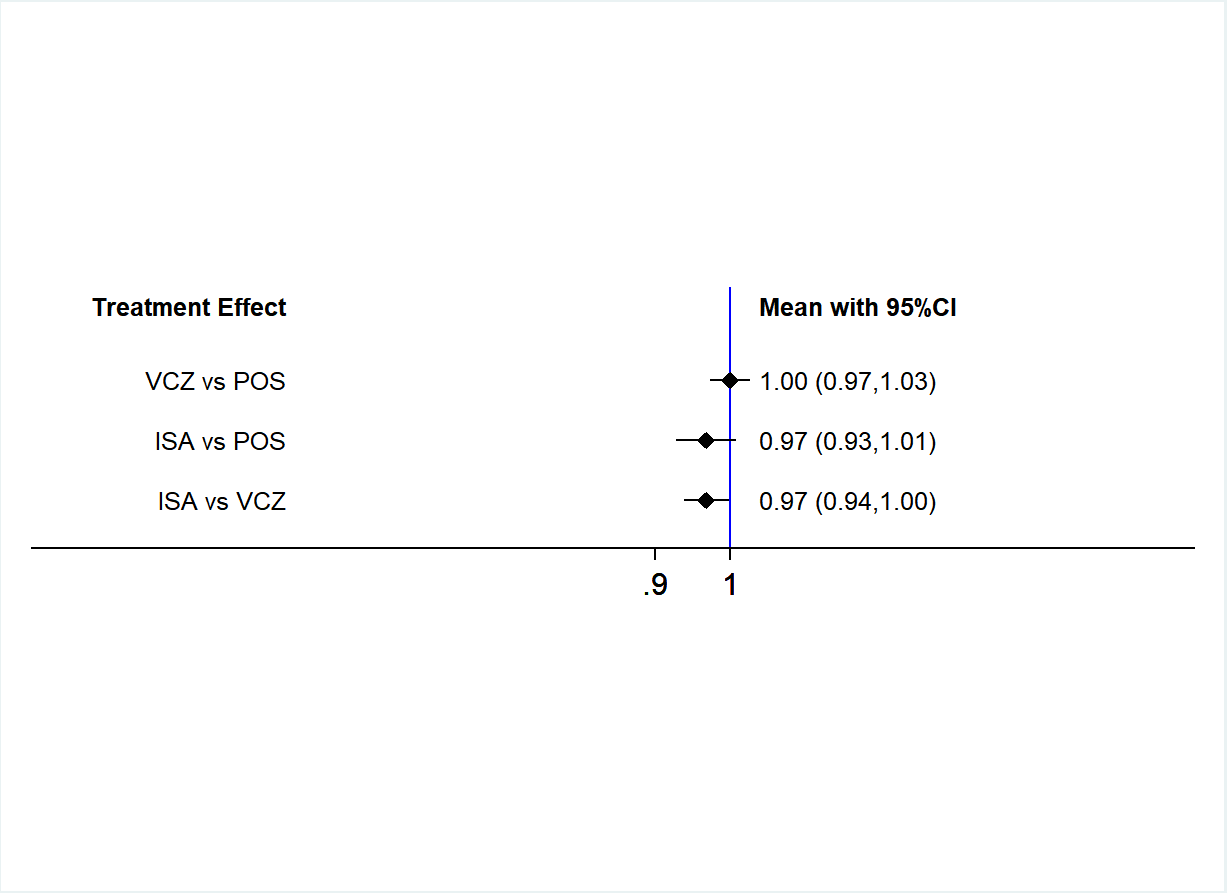


**E.serious adverse events**


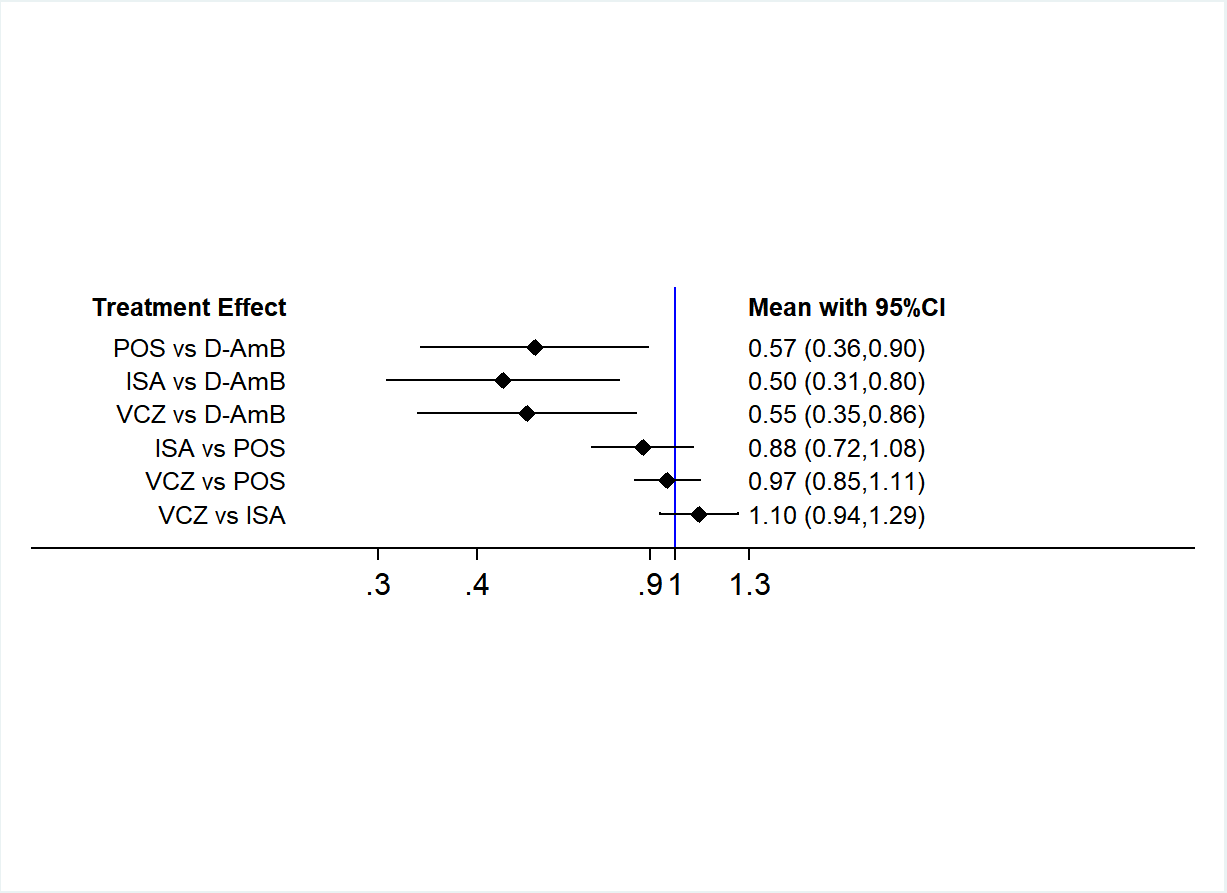


VCZ, voriconazole; D-AmB, amphotericin B deoxycholate; POS, posaconazole; ISA, isavuconazole; RR, relative rare; 95%CI=95% Confidence Interval.
